# Stress management with HRV following AI, semantic ontology, genetic algorithm and tree explainer

**DOI:** 10.1038/s41598-025-87510-w

**Published:** 2025-02-17

**Authors:** Ayan Chatterjee, Michael A. Riegler, K. Ganesh, Pål Halvorsen

**Affiliations:** 1 Oslo Metropolitan University (Oslomet), Oslo, Norway; 2 STIFTELSEN NILU, Kjeller, Norway; 3 Simula Metropolitan Center for Digital Engineering (SimulaMet), Oslo, Norway; 4 School of Mathematics, Indian Institute of Science Education and Research (IISER), Thiruvananthapuram, India

**Keywords:** HRV, Stress, Genetic algorithm, Random forest, Data balancing, SHAP, Semantic ontology, Ethical AI, Health care, Computational science

## Abstract

Heart Rate Variability (HRV) serves as a vital marker of stress levels, with lower HRV indicating higher stress. It measures the variation in the time between heartbeats and offers insights into health. Artificial intelligence (AI) research aims to use HRV data for accurate stress level classification, aiding early detection and well-being approaches. This study’s objective is to create a semantic model of HRV features in a knowledge graph and develop an accurate, reliable, explainable, and ethical AI model for predictive HRV analysis. The SWELL-KW dataset, containing labeled HRV data for stress conditions, is examined. Various techniques like feature selection and dimensionality reduction are explored to improve classification accuracy while minimizing bias. Different machine learning (ML) algorithms, including traditional and ensemble methods, are employed for analyzing both imbalanced and balanced HRV datasets. To address imbalances, various data formats and oversampling techniques such as SMOTE and ADASYN are experimented with. Additionally, a Tree-Explainer, specifically SHAP, is used to interpret and explain the models’ classifications. The combination of genetic algorithm-based feature selection and classification using a Random Forest Classifier yields effective results for both imbalanced and balanced datasets, especially in analyzing non-linear HRV features. These optimized features play a crucial role in developing a stress management system within a Semantic framework. Introducing domain ontology enhances data representation and knowledge acquisition. The consistency and reliability of the Ontology model are assessed using Hermit reasoners, with reasoning time as a performance measure. HRV serves as a significant indicator of stress, offering insights into its correlation with mental well-being. While HRV is non-invasive, its interpretation must integrate other stress assessments for a holistic understanding of an individual’s stress response. Monitoring HRV can help evaluate stress management strategies and interventions, aiding individuals in maintaining well-being.

## Introduction

### Overview

Heart rate variability (HRV) is the difference between heartbeats that serves as a notable indicator of the autonomic nervous system’s adaptability and its regulation of the body’s stress response^1,2^. It gauges the activity of the autonomic nervous system, particularly the balance between its sympathetic and parasympathetic branches^1,2^. HRV has undergone thorough research regarding its correlation with stress, offering valuable insights into an individual’s physiological reaction to stressors^1,2^. Even though these fluctuations are imperceptible other than with special equipment, they can still reveal issues related to health, including heart conditions and mental disorders like anxiety and depression^1,2^. Stress can be described as a mental state of anxiety or tension caused by a difficult situation^3^. Stress is a natural response that humans employ to address threats and challenges in their lives^3^. Overly intense stress can lead to physical and mental issues^3^. Long-term stress can worsen existing health conditions and contribute to increased use of alcohol, tobacco, and other substances. Additionally, stressful situations can exacerbate mental health issues, notably anxiety and depression, underscoring the importance of access to healthcare services^3^. Individuals vary in their coping mechanisms and the symptoms they experience when under stress. Over time, many people find that stress levels diminish as the situation improves or as they develop effective emotional coping strategies^3^.

HRV typically can be measured using electrocardiography (ECG)^4^ or photo-plethysmography (PPG)^5,6^. ECG^4^ involves attaching electrodes to the chest, typically in a three or five-lead configuration, to record the electrical signals produced by the heart. On the other hand, PPG^5,6^ functions by emitting light into the skin, typically on the fingertip or earlobe, and measuring the volume of blood reflected to the sensor is based on this light. Once the HRV signal is obtained using either ECG or PPG, it undergoes several processing steps to extract relevant features and calculate HRV metrics^7^. HRV measurement and analysis can be influenced by factors such as the duration of the recording, the posture of the individual, and the specific analytical techniques used^8,9^. Therefore, a standard process and guidelines must be followed to ensure consistent and reliable HRV assessment. Several publicly accessible datasets are available for use in machine learning research that involves HRV, such as Physio Net HRV Databases^10^, European ST-T Database^11^, Physio Bank Normal Sinus Rhythm Database (nsr2db)^12^, Short-Term HRV Database^13^, MIMIC-III Database^14^, Database for Emotion Analysis using Physiological Signals (DEAP)^15^, Wearable Stress and Affect Detection DataSet (WESA)^16^, MAHNOB-HCI multimodal database^17^, Multi-Modal Stress Dataset (MMSD)^18^, University of Waterloo Stress Dataset (UWS)^18^, and Rodbound University SWELL-KW dataset^19^.

Machine learning algorithms have been applied in HRV data analytics across various studies to uncover complex patterns, perform feature engineering, and predict stress levels. Feature engineering^20^ involves selecting relevant features from the dataset to improve model performance and computational efficiency. By focusing on key features, researchers can develop more accurate, streamlined, and interpretable models, facilitating the extraction of valuable insights from the data. This process simplifies models, identifies critical features, reduces bias, and enhances transparency and trust, aligning with the principles of explainable AI (X-AI)^21,22^. X-AI techniques^23,24^ address the opacity of complex models, bridging the gap between their “black box” nature and the necessity for transparency and comprehensibility. Feature selection, X-AI, bias, fairness, and ethical AI are interconnected concepts essential for building responsible and unbiased machine learning models^25,26^. Additionally, addressing data imbalances through techniques like data balancing^27^, can mitigate model biases arising from skewed class distributions in training data. Nonetheless, it’s crucial to acknowledge and mitigate other potential sources of bias to ensure fairness and impartiality in predictions.

The concept of Ethical AI in health prediction models underscores the importance of patient privacy, adherence to data protection laws, and the mitigation of bias and discrimination^26,28–32^. It acknowledges that biases can stem from various sources, such as biased training data or flawed model assumptions, resulting in inaccurate or unjust predictions. Ethical AI necessitates careful consideration of data collection, evaluation metrics, and model fairness to ensure unbiased predictions across different demographic groups. Transparency is crucial, as it offers explanations for model predictions, enhancing comprehension among healthcare professionals and patients, fostering trust in AI systems, and facilitating error identification and rectification. Additionally, Ethical AI prioritizes rigorous verification and testing of predictions to minimize false outcomes and mitigate harm to patients. By prioritizing safety and well-being, Ethical AI endeavors to provide accurate and dependable health predictions. Regular evaluation of model performance, impact, and fairness, coupled with continuous analysis of ethical implications, supports ongoing refinement and optimization of models while upholding ethical standards

### Motivation

The motivation behind this study lies in the critical role of HRV as an indicator of the autonomic nervous system’s adaptability and its regulation of the body’s stress response. HRV offers valuable insights into an individual’s physiological reaction to stressors, aiding in stress management and the prevention of associated mental health problems like anxiety and depression. With stress being a pervasive aspect of modern life, understanding and monitoring it are crucial for maintaining overall well-being. Therefore, this study aims to delve into HRV-based stress prediction using machine learning techniques, focusing specifically on the SWELL-KW dataset. This study focuses on leveraging machine learning techniques to develop accurate and interpretable models for stress prediction using HRV data, while also addressing important research questions related to feature extraction, model construction, result interpretation, ethical compliance, and semantic knowledge representation.

### Aim of the study

The primary aim of this study is to devise a machine-learning pipeline capable of accurately classifying multi-class stress levels using HRV data. By leveraging meta-heuristic feature selection methods^33^, traditional and ensemble classifiers^34^, and calibrated techniques^35^, the study seeks to enhance model performance while mitigating issues such as overfitting, bias, and variance. Furthermore, the study aims to evaluate the effectiveness of the developed pipeline on both imbalanced and balanced datasets to discern any disparities in results. Additionally, the study emphasizes the importance of model transparency and ethical compliance, utilizing model explanation tools to ensure interpretability and accountability. Through rigorous evaluation and comparison with existing methodologies, the study aims to address key research questions related to feature extraction, model construction, result communication, ethical considerations, and semantic knowledge representation in the context of HRV-based stress prediction. In a broader sense, the identified research questions (RQs) for this theoretical and conceptual study are : 

- How can feature extraction be conducted when dealing with non-linear features within a dataset?
- What strategies can be employed to construct and implement a machine learning pipeline capable of effectively analyzing both imbalanced and balanced datasets?
- In what manner can predictive analysis results be communicated meaningfully?
- How can the ethical compliance and responsibility of the adopted approach be articulated?
- What methods can be utilized to semantically represent the acquired knowledge substantially?

Selecting a specific case study has streamlined this study’s focus on addressing the research questions. Yet, there’s potential for expansion by incorporating additional HRV datasets akin to the chosen one. The research underscores the significance of HRV data in stress management, particularly when integrated with an ontology-based method. Stress, a key factor in mental health issues like depression, necessitates ongoing surveillance to avert related mental health challenges in individuals.

### Study distribution

The remainder of the study is formulated as follows. The *Related Work* Section describes the existing studies and our contributions. The *Methods* Section describes the adopted methodology for this study, such as ethical approval, data collection strategy, the description of collected data, feature engineering and data balancing strategies, selection and description of machine learning classifiers, description of classification explanation models, machine learning model evaluation strategies, problem formulation, and the description of the proposed machine learning pipeline relevant for the problem and to answer the research question. The *Result* Section elaborates on feature selection, ontology model, and classification outcomes. The *Discussion* Section describes the relevance of the study results with classification explanations and ethical AI approaches. The study is concluded in the *Conclusion* Section.

## Related work

HRV can be measured using different sensors, such as a full ECG, more practical devices like chest straps (e.g., Polar H7 or H10), or devices utilizing optical technology (PPG)^36^. Wearable devices commonly use PPG, employing either green LEDs (visible to the bottom of the sensor, as seen in Garmin watches or Whoop bands) or infrared light (e.g., Oura)^36^. Several studies have utilized various HRV datasets for stress level classification, employing standard feature selection techniques followed by machine learning models. Muhajir et al.^37^ achieved 70$\%$ accuracy on linear analysis and 60$\%$ accuracy on non-linear analysis using sixteen HRV features. Giannakakis et al.^38^ utilized sixteen features with an SVM classifier and achieved 84.4$\%$ accuracy with transformed features. Liu et al.^39^ employed Spearman rank correlation and Bland-Altman plots analysis for feature selection, achieving 85.3$\%$ accuracy with an SVM classifier. Can et al. collected self-reports and physiological measures, obtaining 73.44$\%$ accuracy with the SVM classifier. Castaldo et al.^40^ used different machine-learning models and obtained 80.0$\%$ accuracy for stress detection from linear and non-linear HRV features. Hovsepian et al.^41^ predicted stress using ECG and respiration data, achieving 90.0$\%$ and 72.0$\%$ accuracy in laboratory and real-life settings, respectively, with the SVM classifier. Gjoreski et al.^42^ built a stress detector with a wearable wrist device, achieving 92.0$\%$ accuracy with the SVM classifier. Schmidt et al.^43^ classified HRV features towards stress detection, achieving 88.0$\%$ accuracy with the Random Forest (RF) classifier. Muaremi et al.^44^ predicted stress using multiple biomarkers, achieving 71.0$\%$ accuracy with the Random Forest (RF) classifier. Benchekroun et al.^45,46^ conducted a cross-dataset analysis, achieving an F1 score of 71.0$\%$ with the Logistic Regression (LR) classifier on the MMSD dataset and an F1 score of 75.0$\%$ with the Random Forest (RF) on the UWS dataset.

Further studies have addressed stress classification tasks using various datasets and machine learning techniques. Sriramprakash et al.^47^ focused on a binary classification task with the SWELL-KW dataset, achieving a 92.7$\%$ accuracy score using an SVM classifier with 17 features. Sarkar et al.^48^ tackled multi-class classification problems with SWELL-KW and AMIGOS datasets, achieving 98.3$\%$ accuracy with a Convolutional Neural Network (CNN). Koldijk et al.^49^ also worked on multi-class classification using the SWELL-KW dataset, achieving 90.0$\%$ accuracy with an SVM classifier. Bobade et al.^50^ conducted binary and multi-class classification on the WESAD dataset using machine learning classifiers, such as Artificial Neural Networks (ANNs), obtaining accuracy scores of 84.32$\%$ and 95.21$\%$, respectively, with seven features. Similarly, Arsalan et al.^51^ performed binary and multi-class classification on WESAD using Multi-Layer Perceptrons (MLPs), achieving 92.85$\%$ and 64.28$\%$ accuracy, respectively, with seven features. Albaladejo et al.^52^ worked on a binary classification task with the SWELL-KW dataset, reaching an 88.64$\%$ accuracy score using an MLP classifier with all 34 features. Mortensen et al.^53^ addressed multi-class classification with SWELL-KW, achieving 99.99$\%$ and 96.5$\%$ accuracy scores with a 1D-CNN classifier using all 34 features and an optimized set of 15 features, respectively. They utilized the Analysis of Variance (ANOVA) method for feature optimization. Additionally, Ghose et al.^54^ explored HRV features as potential biomarkers for stress detection, achieving a 99.3$\%$ accuracy score with three features using the k-nearest neighbors (k-NN) algorithm.

Feature reduction in machine learning can enhance model accuracy and performance, but it also presents potential drawbacks, including information loss, overfitting, bias, oversimplification, failure to capture complex relationships, and model instability. The decision to reduce features should be informed by a comprehensive understanding of the data and its nuances. Meticulous attention to design, data analysis, and feature validation is vital to prevent diminishing model accuracy or reducing generalizability. This study extends previous work by focusing on the following things and a comparative qualitative study has been captured in Supplementary Material-1  : 

- Utilizing meta-heuristic algorithms for feature optimization to create a relevant feature set, particularly for non-linear features,
- Employing standard oversampling techniques to balance data and conducting classification on both imbalanced and balanced datasets to ensure model fairness,
- Explaining classification outcomes using a tree explainer method,
- Addressing ethical considerations in AI applications, and
- Implementing semantic representation to efficiently manage both data and knowledge derived from the study.

## Methods

The methodology section outlines the approach used in the study. It covers the data collection and feature extraction processes, the machine learning classification and explanation models considered, metrics for model evaluation, experimental setup, and the necessity of ethical approval. Adhering to the General Data Protection Regulation (GDPR) guidelines^55^, the study ensures compliance with data collection and governance standards.

### Data description and feature relationship

The SWELL-KW dataset^19,56^ is a comprehensive collection encompassing a range of data types, including HRV, body positions, facial expressions, and skin conductance. These diverse data types offer insights into both behavioral and physiological aspects. The dataset originates from an experiment involving 25 participants engaged in typical knowledge-related tasks under stressful conditions, such as time pressure and email interruptions. Data was captured through various means, such as computer logging (screen logs), facial expressions, body postures, ECG, and skin conductance, during both neutral and stressful scenarios. With 34 features and 1 predicting class, the dataset presents an imbalance in class distribution across its 410,322 records. Despite this, the dataset is deemed clean, and devoid of any null or NAN values. To handle the categorical nature of the predicting class, a label encoding approach was employed, resulting in class representations as 0, 1, and 2. The features of the dataset are explained by Mortensen et al.^53^. The dataset has the following three classes for predictive analysis-

- Interrupt (**0**): The participants were given 8 emails during the middle of their assigned duties. Some emails were pertinent to their duties, and the participant was requested to perform specific actions on them, while others were simply not pertinent to their duties.
- No stress (**1**): The subjects are permitted to engage in the tasks for as long as they needed for a maximum of 45 minutes, but they were not aware of the maximum length of their tasks.
- Time pressure (**2**): During this period, the participant was required to finish the task within 2/3 of the time required in the neutral condition.

**Figure 1:**
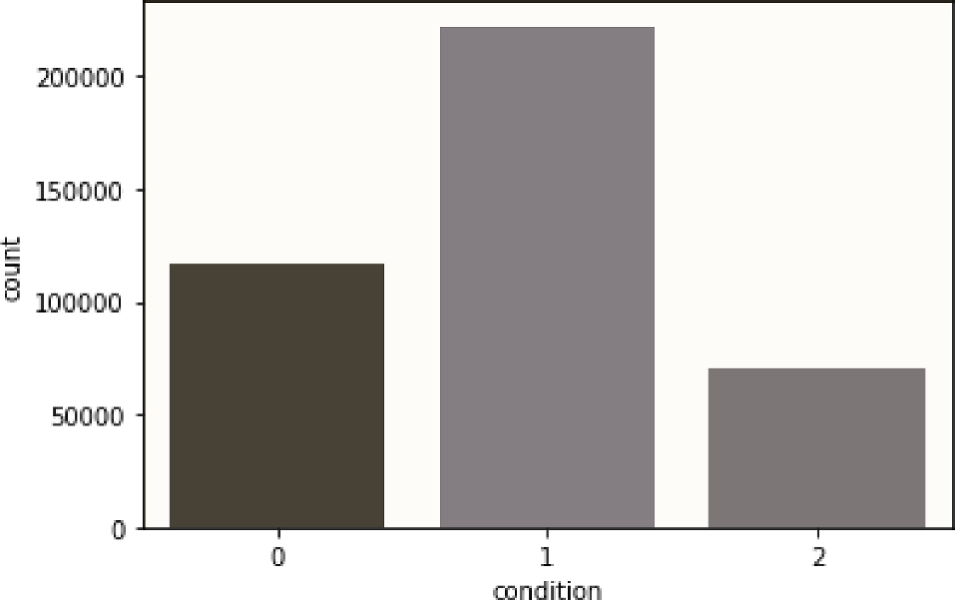
The distribution of the labeled SWELL-KW dataset with a Label Encoder.

Non-linear data relationships occur when a linear model fails to adequately describe the connection between variables. Understanding such relationships often requires exploratory data analysis, visualizations, and domain knowledge. Several techniques address non-linearity: Polynomial Regression, Linear and Non-linear Regression, Decision Trees, Kernel methods (e.g., Support Vector Machines, Gaussian Processes), Neural Networks, Ensemble Methods, and Non-parametric methods (like k-nearest neighbors, locally weighted regression). In our study, we utilized Linear Regression despite the presence of non-linear relationships. We split the dataset into training and testing sets, training Linear and Polynomial Regression models (with degree 2) on the former. Subsequently, we evaluated the models on the testing set, assessing their performance using the R-squared metric. An R-squared value greater than 0.8 suggests linear dependence, while the values below indicate non-linear dependency. Furthermore, we excluded the detailed description of the HRV features as they are available in the existing literature^38,57–62^ and online web^63,64^. Their labeled distribution has been depicted in Fig. 1.

Initially, we started with the original (imbalanced) HRV dataset and employed all the classification models on the following four different formats of the datasets. Then, we applied feature engineering to the most relevant format of the data, including the original forms^65^, such as MinMaxScaler(feature$\_$range=(0,1), Normalizer(norm=’l1’), Normalizer(norm=’l2’), and Standard Scaler.

### Data balancing

Class imbalance^66–70^ in datasets can lead to biased models that prioritize the majority class, resulting in poor performance on the minority class. This imbalance can also hinder learning, as insufficient examples of the minority class may prevent the model from capturing important patterns. Moreover, it can contribute to overfitting, where the model becomes overly specialized in the majority class and struggles to generalize to new data. Additionally, the class imbalance can exacerbate concept drift, as changes in the minority class may go unnoticed due to its limited representation. Different data-balancing techniques have been proposed in machine learning literature, including Random Oversampling, Random Undersampling, SMOTE, and ADASYN. These methods aim to address class imbalance by generating additional samples for the minority class in regions of the feature space where it is underrepresented.

This study employs the SMOTE^27,71^ and ADASYN^72^ algorithms for data labeling and incorporates stratification and shuffling techniques during data partitioning for training, validation, and testing. Stratification, a data sampling method, is utilized to maintain the distribution of classes in the generated subsets. SMOTE works by creating synthetic instances within the feature space of the minority class, interpolating between existing minority class samples. ADASYN is an enhancement to SMOTE, designed to handle data with intricate class distributions more effectively. While SMOTE generates synthetic samples by oversampling the minority class, ADASYN adjusts this process based on the local density distribution of the minority class. This adaptive approach aims to better represent the underlying data distribution and provides a more robust solution for addressing class imbalance. The algorithmic steps of SMOTE and ADASYN are outlined in Algorithm 1 and Algorithm 2. Unlike SMOTE, which produces the same number of synthetic samples for all minority classes, ADASYN dynamically alters the number of synthetic samples generated for each instance based on its local neighborhood. This adaptability makes ADASYN particularly useful for datasets with varying degrees of class imbalances and complex class distributions.

**Algorithm 1:**
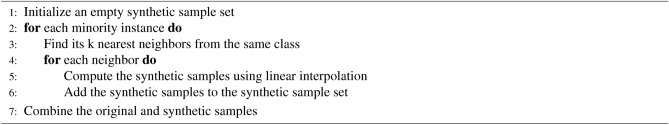
SMOTE Algorithm

**Algorithm 2:**
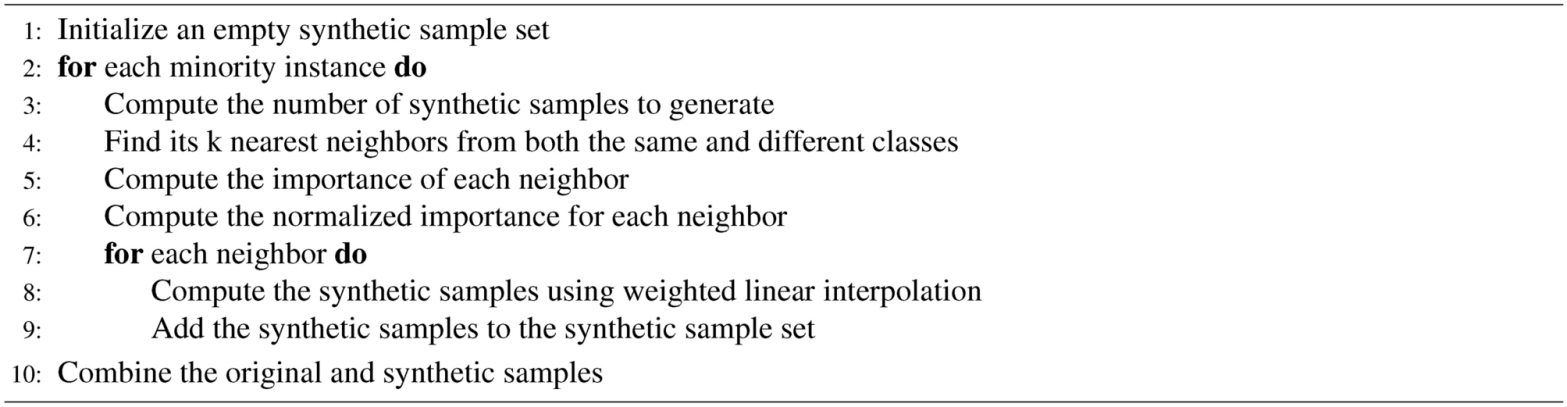
ADASYN Algorithm

### Feature engineering

Feature engineering creates an optimal feature set or changes the way existing features are incorporated into a dataset to enhance the performance of machine learning models^73–76^. It’s important to take away the complexities from the raw data and present it in a way that is more conducive to the learning algorithm. This process plays a pivotal role in improving model performance, reducing overfitting, enhancing model interpretability, reducing dimensionality, increasing efficiency, and fostering robustness and versatility to changing data. Feature optimization allows the learning algorithm to leverage the most informative aspects of the data, leading to more accurate and legitimate predictions or classifications^73–76^. The standard feature selection methods are described in Supplementary Material-2.

In analyzing the SWELL-KW dataset, characterized by its non-linear feature relationships, we employed the Genetic Algorithm (GA) to optimize features and assess resulting dataset performance across various Machine Learning (ML) classifiers. Our approach included a comparative evaluation of GA-based feature optimization against alternative feature selection methods. By iteratively executing selection, reproduction, and replacement processes, the genetic algorithm navigates solution spaces, progressively converging toward more effective solutions. The algorithm operates on the principle of favoring the survival of the fittest, facilitating the propagation of genetic material with higher fitness levels. Utilizing *sklearn-genetic*^77^, key parameters such as cross-validation size (e.g., 5), scoring, population size, number of generations, mutation rate or probability, crossover independent probability, mutation independent probability, and tournament size were specified for feature selection. The **GA** has been represented in Algorithm 3.

**Algorithm 3:**
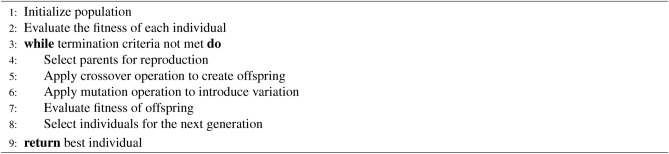
Genetic Algorithm (GA)

### Machine learning classification models

We utilized standard ML algorithms for classification tasks, integrating 5-fold cross-validation, a random state of 42, and Grid-search for hyperparameter optimization. These methods were selected due to their prevalence in supervised ML tasks and their effectiveness with medium-sized datasets, particularly for binary classification^78–81^—Logistic Regression (LR), Linear Discriminant Analysis (LDA), K-Nearest Neighbors (KNN), Naïve Bayes (NB), Decision Tree (DT), Random Forest (RF), Bagging Classifier, and Gradient Boosting (GB) Classifier. We have chosen ML classifiers over deep learning classifiers due to several advantages. Firstly, ML classifiers utilize convex optimization techniques in gradient descent to find global minima efficiently. Secondly, they require a smaller amount of training data compared to deep learning models. Thirdly, ML models have minimal training time, making them suitable for scenarios with time constraints. Fourthly, ML models can be trained on a central processing unit (CPU), eliminating the need for specialized hardware. Fifthly, they are computationally inexpensive and occupy little storage space. Finally, ML models are transparent, allowing for easier interpretation of results. Support Vector Classifier (SVC) was excluded due to its high computational complexity, both with and without kernels, making it impractical for our purposes.

Additionally, we employed CalibratedClassifierCV to calibrate the predicted probabilities of our classification model. Calibration is crucial in real-world scenarios where accurate probability estimates are necessary for decision-making. CalibratedClassifierCV fits a calibration model on the output of the base classifier, refining the predicted probabilities to be more reliable and interpretable as actual probabilities. This enhancement ensures better confidence level-based decision-making. Overall, the adopted steps for the machine learning classification are as follows in the form of Algorithm 4, and the used ML models are described in Supplementary Material-3. Ensemble methods in machine learning offer a distinct approach by amalgamating multiple individual models to construct a more robust and precise predictor. This strategy capitalizes on the principle that aggregating predictions from diverse models can yield superior results compared to relying on any single model alone. The ensemble classification process is delineated in detail through Algorithm 5. The machine learning models employed in this methodology are thoroughly expounded upon in Supplementary Material-3.

**Algorithm 4:**
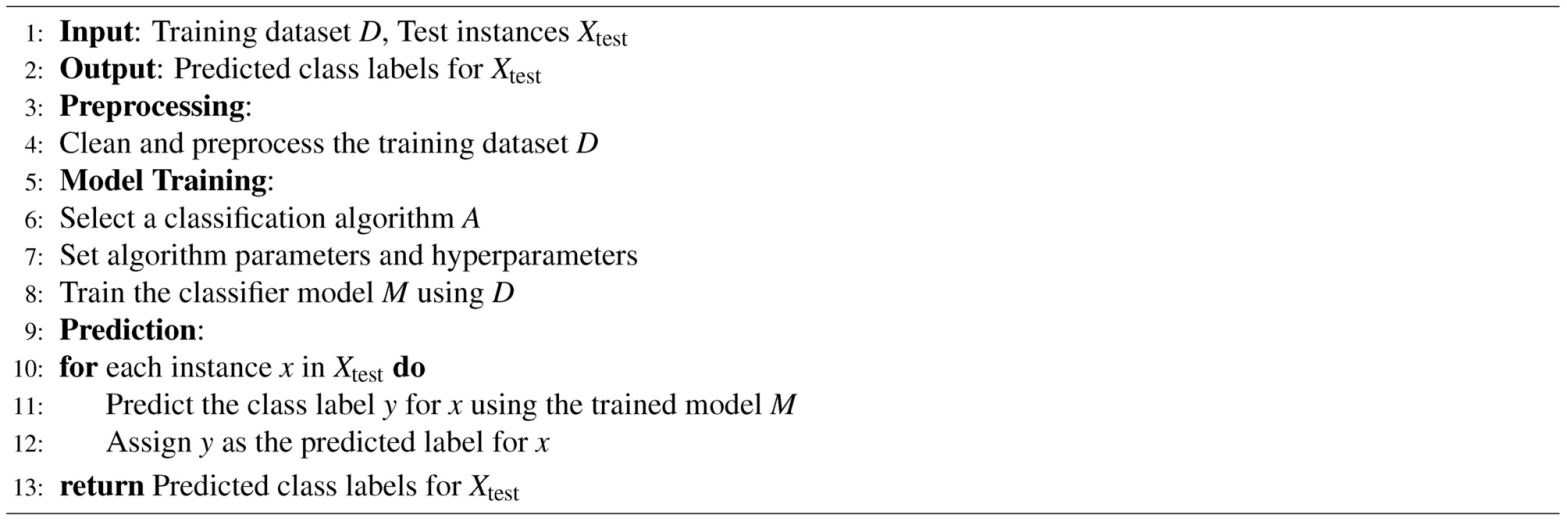
Machine Learning Classification

**Algorithm 5:**
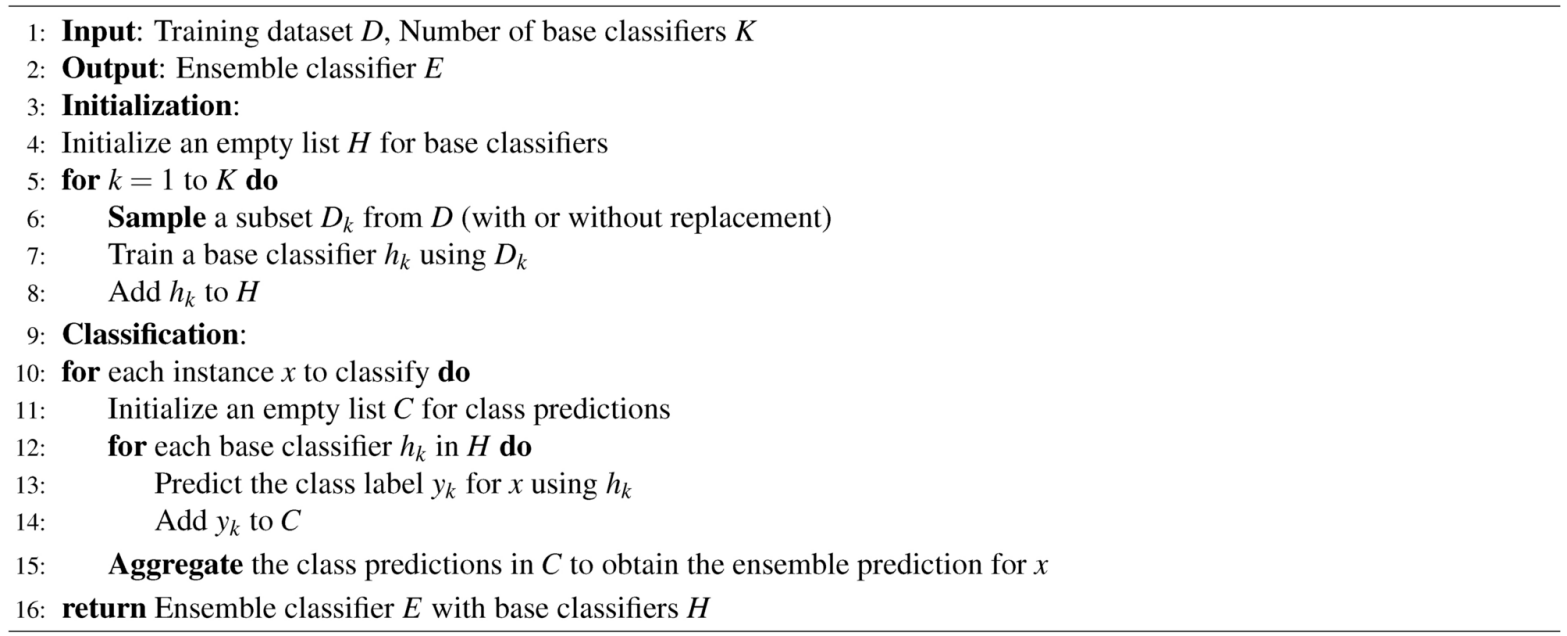
Ensemble Classification

The choice of algorithm for a given task hinges on several factors such as the problem’s nature, dataset size, data type, and desired performance. Experimentation with various algorithms is often advantageous to assess their efficacy for a specific problem. However, in the context of multi-class classification with a medium-sized dataset, certain classifiers stand out due to their simplicity, transparency, and ability to handle nonlinear data. In our classification approach, we initially load the labeled dataset and partition it into dependent and independent features. Subsequently, we construct a classification model, training it against an optimal number of features. Evaluation of the model is conducted using five-fold cross-validation, employing ’accuracy’ as the scoring metric. Hyperparameter tuning is performed utilizing the grid-search method to enhance model performance. The effectiveness of the selected classification models is then compared. The final prediction of ML models is derived by averaging the predictions of individual models for regression tasks or through majority voting for classification tasks. Bagging (e.g., RF) effectively mitigates variation and overfitting by incorporating multiple, diverse models. Table 1 describes different models and their hyperparameters to be tuned using the GridSearchCV method.

**Table 1:** The parameter list of the considered ML models for hyperparameter tuning.

Model	Parameters
LR	’penalty’: [’l1’, ’l2’], ’$\alpha$’: [0.1, 1, 10], ’solver’: [’liblinear’, ’saga’], ’max$\_$iter’: [100, 200, 500]
LDA	’solver’: [’svd’, ’lsqr’, ’eigen’], ’shrinkage’: [None, ’auto’, 0.5], ’n$\_$components’: [None, 1, 2, 3]
RF	’criterion’: [’gini’, ’entropy’], ’max$\_$depth’: [2, 4, 6, 8], ’n$\_$estimators’: [100, 200, 300]
DT	’criterion’: [’gini’, ’entropy’], ’max$\_$leaf$\_$nodes’: [2, 4, 6, 8, 10, 12], ’min$\_$samples$\_$split’: [2, 3, 4]
NB	’var$\_$smoothing’: [1e-9, 1e-8, 1e-7, 1e-6, 1e-5]
KNN	’n$\_$neighbors’: [2, 4, 6], ’weights’: [’uniform’, ’distance’], ’p’: [1, 2]
Bagging	’n$\_$estimators’: [50, 100, 200], ’max$\_$samples’: [0.5, 0.7, 1.0], ’max$\_$features’: [0.5, 0.7, 1.0], ’min$\_$samples$\_$split’: [2, 5, 10]
GB	’$\alpha$’: [0.1, 0.01, 0.001], ’n$\_$estimators’: [50, 100, 200], ’max$\_$depth’: [3, 5, 7], ’min$\_$samples$\_$split’: [2, 5, 10], ’max$\_$features’: [’sqrt’, ’log2’]
AdaBoost	’$\alpha$’: [0.1, 0.01, 0.001], ’n$\_$estimators’: [50, 100, 200], ’max$\_$depth’: [3, 5, 7], ’min$\_$samples$\_$split’: [2, 5, 10], ’max$\_$features’: [’sqrt’, ’log2’]

### Classification explanation model

We utilized the SHAP tree-based explanation model^81–84^ to elucidate the feature importance within the context of classification. Renowned for its popularity, consistency, comprehensiveness, and reliability, SHAP has emerged as a widely embraced method for interpreting tree-based models. Its adoption has notably enhanced the interpretability and transparency of machine learning across diverse fields. The SHAP algorithm has been expressed in Algorithm 6.

**Algorithm 6:**
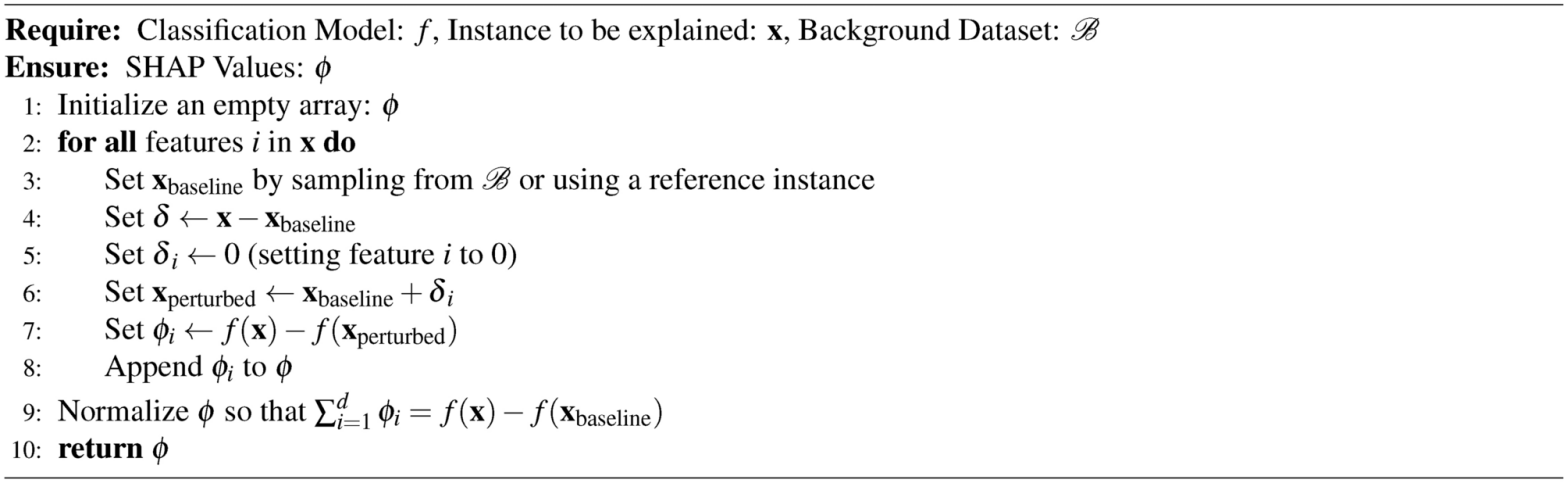
SHAP Explanation Algorithm

SHAP visualization is integral for enhancing the interpretability and explainability of classification models. It empowers users by offering profound insights into the decision-making mechanism of the model, thereby bolstering confidence in AI systems and facilitating informed decision-making based on model outcomes. The utilization of color-coded representations to depict feature contributions in SHAP interpretations serves to enhance visual clarity, rendering the model’s decision process more comprehensible. Through this visualization, one can elucidate how each feature influences the model’s prediction for a specific instance, with color coding aiding in discerning positive, negative, and negligible contributions. The SHAP value $\phi _i$ represents the contribution of that feature to the model’s prediction in the following way-

- If $\phi _i > 0$, the feature *i* has a positive contribution to the prediction, pushing it higher. We can use red color to represent positive contributions.
- If $\phi _i < 0$, the feature *i* has a negative contribution to the prediction, pushing it lower. We can use green or blue colors to represent negative contributions.
- If $\phi _i = 0$, the feature *i* does not significantly impact the prediction for this instance. We can use gray color to represent negligible contributions.

SHAP values ($\phi _i$) can be effectively visualized through either a bar chart or a summary chart. In both visualizations, each feature is depicted by a bar, with the length of the bar representing its corresponding SHAP value. Additionally, the color of the bars aligns with the color code established earlier, providing clarity regarding the post type associated with each feature.

### Classifier evaluation metrics and metric relationships

Different metrics assess classification performance, including accuracy, precision, recall, F1 score, and Mathew’s correlation coefficient (MCC)^85,86^. Accuracy gauges the proximity of measurements to actual values, while precision evaluates the relative accuracy of measurements. Recall measures the total positive results returned by a machine learning model. However, accuracy and F1 scores can be misleading as they require a comprehensive account of the confusion matrix’s four categories for accurate score calculation. MCC surpasses the F1 score and accuracy in informativeness by considering the balance among true positives, true negatives, false positives, and false negatives within the confusion matrix^85,86^.

1 $$\begin{aligned} Precision = \frac{TP}{TP + FP} \end{aligned}$$

2 $$\begin{aligned} Recall = Sensistivity = \frac{TP}{TP + FN} \end{aligned}$$

3 $$\begin{aligned} Accuracy = \frac{TP+TN}{TP+TN+FP+FN} \end{aligned}$$

4 $$\begin{aligned} F1\text {-}score = \frac{2 \times Recall \times Precision}{Recall+Precision} \end{aligned}$$

5 $$\begin{aligned} \small MCC = \frac{TP*TN - FP*FN}{\sqrt{(TP+FP)(TP+FN)(TN+FP)(TN+FN)}} \end{aligned}$$

The evaluation metrics-accuracy, precision, recall, F1 score, and MCC-are closely related and together provide a comprehensive understanding of a model’s performance. Accuracy measures the proportion of correctly classified samples out of all samples, making it intuitive but less informative for imbalanced datasets. Precision reflects the proportion of true positive predictions among all positive predictions, reflecting significance. Recall measures the ability to capture all true positives, reflecting sensitivity. F1 score combines precision and recall into a single value to better handle class imbalance than accuracy alone. MCC stands out as a balanced metric, encompassing all elements of the confusion matrix to evaluate performance, even in imbalanced scenarios.

### Machine learning pipeline design

**Figure 2:**
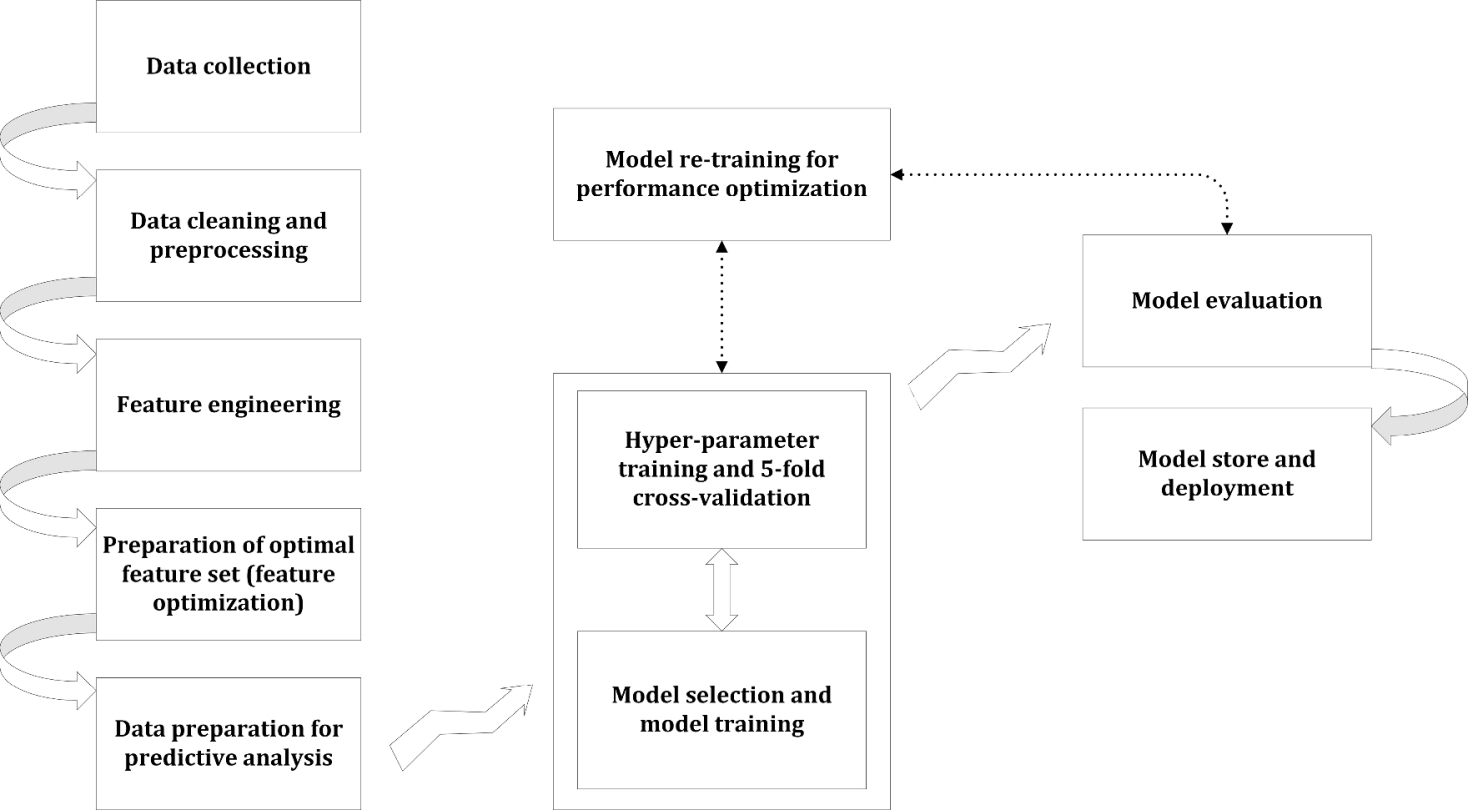
The high-level structure of the adopted machine learning pipeline.

A well-designed machine learning pipeline enhances productivity, consistency, and collaboration in model creation and distribution^87,88^. It streamlines the process, reduces errors, and boosts model quality and reliability. However, due to the iterative nature of machine learning, pipeline steps may require revisiting or refining based on data insights or model performance. Hence, pipeline design should be flexible to accommodate adjustments and enhancements. Typically, a machine learning pipeline comprises multiple sequential steps, each serving distinct purposes. The design must be adaptable to incorporate changes and improvements as needed throughout the process. Detailed procedures for a machine learning pipeline are provided in Supplementary Material-4. Overall, Algorithm 7 describes the adopted and summarized steps to build our machine learning classification pipeline. Algorithm 8 informs the high-level steps for hyperparameter tuning in machine learning classification. The high-level structure of the adopted machine learning pipeline has been depicted in Fig. 2.

**Algorithm 7:**
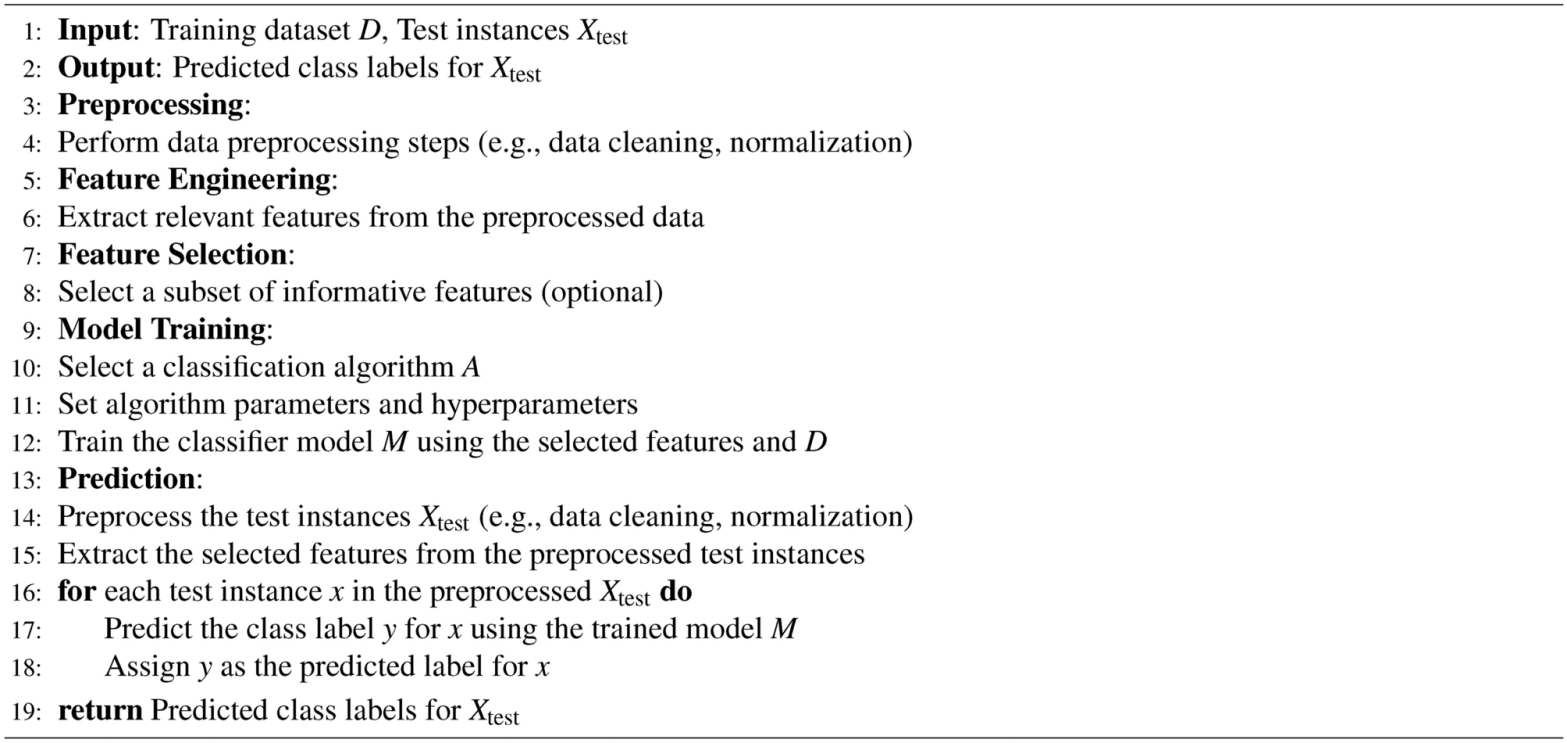
Machine Learning Classification Pipeline

**Algorithm 8:**
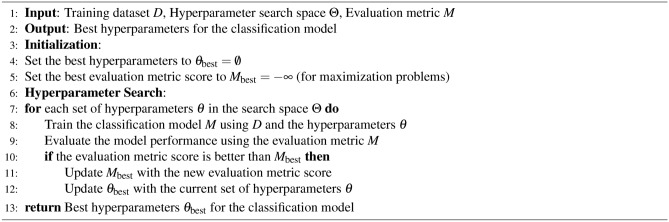
Hyperparameter Tuning for Machine Learning Classification

### Semantic representation of knowledge

Semantic ontology^89–92^ refers to the structured representation of knowledge using ontologies to articulate and organize information in a manner that encompasses semantics, meaning, and relationships among concepts. It serves as a robust framework for representing, organizing, and reasoning with knowledge, taking into account the semantics and intended meaning of the information. Semantic ontologies facilitate advanced reasoning abilities, interoperability, and context-appropriate knowledge discovery, thereby enhancing the intelligence and effectiveness of knowledge-based systems and applications. An ontology^89,90^ is a formal and explicit depiction of a shared understanding of a domain, structured in a formalized manner. Comprising a set of concepts (classes) with associated properties (attributes) and connections (associations) between these classes, ontologies typically employ formal languages like the Web Ontology Language (OWL) to represent knowledge in a machine-readable form. Knowledge representation through ontology offers a systematic and linear approach to acquiring, organizing, and reasoning with domain knowledge, enabling richer and more expressive representations compared to traditional databases.

Ontologies are generally domain-independent and reusable across diverse applications and domains, facilitating interoperability and data integration. They support reasoning and automatic logical consistency checking within the knowledge base, capturing the semantics of concepts and relationships to enhance data understanding and processing. Ontologies provide a common vocabulary for systems and applications to communicate effectively (semantic and structural interoperability), simplifying tasks such as information retrieval, classification, and reasoning. In contrast, database schemas are primarily designed for efficient data storage, retrieval, and manipulation, defining the structure of tables, columns, and relationships between tables for data organization and management^89,90^. We have designed and developed an ontology mapping for the semantic annotation (data and knowledge representation) to represent all the features and facilitate knowledge sharing and reuse, data integration, reasoning, and query with a query language. In the object-oriented representation, owl:Thing acts as a global parent class and the arrows define a hierarchical relationship (IS-A) between the concepts. We have defined an ontology as a set $\mathcal {O}$ consisting of classes *C*, relations *R*, and instances *I*. It then specifies the class hierarchy using the relation $\text {subclassOf}$, the relation hierarchy using $\text {subpropertyOf}$, and the instance-of relationship using $\text {instanceOf}$.

**Ontology**

$$\mathcal {O} = \{C, R, I\}$$

**Classes:**

$$C = \{c_1, c_2, \ldots , c_n\}$$

**Relations:**

$$R = \{r_1, r_2, \ldots , r_m\}$$

**Instances:**

$$I = \{i_1, i_2, \ldots , i_k\}$$

**Class Hierarchy:**

$$\text {Let } \text {subclassOf}(c_i, c_j) \subseteq C \times C \text { represent the subclass relationship between classes.}$$

**Relation Hierarchy:**

$$\text {Let } \text {subpropertyOf}(r_i, r_j) \subseteq R \times R \text { represent the subproperty relationship between relations.}$$

**Instance-Of Relationship:**

$$\text {Let } \text {instanceOf}(i, c) \subseteq I \times C \text { represent the instance-of relationship between instances and classes.}$$

While traditional ontologies, such as those based on RDF or OWL, may lack native support for complex production rules commonly found in rule-based systems, this limitation is not inherent to ontologies themselves; rather, it reflects a difference in their intended use cases. When an application necessitates efficient rules or intricate reasoning, relying solely on an ontology may not be the most suitable approach. Thus, this study advocates for a hybrid methodology that integrates ontologies with rule bases (knowledge bases) to devise an effective recommendation generation method for managing the complex knowledge base of modeling capabilities. This combination has the potential to mitigate the shortcomings of using ontologies alone. The ontology design and development process utilized Protégé (v. 5.6.1), an open-source platform renowned for ontology editing. Protégé supports the Web Ontology Language (OWL) and offers direct connections to reasoning engines like HermiT and Pellet. HermiT was employed in this study for consistency checking of the developed OWL ontology model, which was subsequently stored in the TTL format.

### Ethical approval

In this project, we have used the publicly available anonymous HRV (SWELL-KW) dataset as a study case. Therefore, no ethical approval has been required.

## Results

This section describes the experimental setup and overall results obtained from the dataset.

### Experimental setup

We conducted our experiments using Colab, also known as Google Colaboratory, a cloud-based Jupyter Notebook environment provided by Google. The computational resources available in Colab, including memory, vary depending on whether you opt for CPU or GPU runtime and the specific hardware configuration selected. Dataset storage requirements are influenced by factors such as dataset size, feature count, and data type. Typically, datasets that can fit within the available memory are efficiently processed in Colab. Given that Google Colab provides 12 GB of RAM, it can effectively handle medium-sized datasets for most data science and machine learning tasks. Therefore, for our machine learning classification tasks on a medium-sized dataset, we opted to use the CPU runtime in Colab instead of the GPU.

### Experimental results

This section encapsulates the results derived from data balancing methods, the empirical assessment of feature optimization techniques and machine learning models, and the graphical representation of the acquired insights utilizing an ontology tree.

#### Data balancing

The original SWELL-KW dataset (**D1**) has 410,322 rows; however, it is imbalanced. The SMOTE data balancing algorithm produced a dataset (**D2**) of 666,720 rows (62.49$\%$ data augmented) and the ADASYN data balancing algorithm produced another dataset (**D3**) of 666,861 rows (62.52$\%$ data augmented). The ADASYN is the extended version of the SMOTE algorithm and is advanced. We carried out our experiments on **D1**, **D2**, and **D3**. The generation of **D2** and **D3** maintained data privacy.

#### Feature selection and semantic representation

The original SWELL-KW dataset has the following 34 features—[’MEAN$\_$RR’, ’MEDIAN$\_$RR’, ’SDRR’, ’RMSSD’, ’SDSD’, ’SDRR$\_$RMSSD’, ’HR’, ’pNN25’, ’pNN50’, ’SD1’, ’SD2’, ’KURT’, ’SKEW’, ’MEAN$\_$REL$\_$RR’, ’MEDIAN$\_$REL$\_$RR’, ’SDRR$\_$REL$\_$RR’, ’RMSSD$\_$REL$\_$RR’, ’SDSD$\_$REL$\_$RR’, ’SDRR$\_$RMSSD$\_$REL$\_$RR’, ’KURT$\_$REL$\_$RR’, ’SKEW$\_$REL$\_$RR’, ’VLF’, ’VLF$\_$PCT’, ’LF’, ’LF$\_$PCT’, ’LF$\_$NU’, ’HF’, ’HF$\_$PCT’, ’HF$\_$NU’, ’TP’, ’LF$\_$HF’, ’HF$\_$LF’, ’sampen’, ’higuci’]. We used Linear Regression, Random Forest Regressor with max$\_$depth of 10, SelectKBest, PCA, Lasso (L1) with alpha or learning rate ($\alpha$) as 0.1, Ridge (L2) with $\alpha$ as 0.1, and GA to prepare an optimized feature set.

We opted to maintain the Random Forest Regressor-based feature selection approach over the Linear Regression-based method due to its superior performance. Specifically, the Random Forest Regressor yielded a root-mean-squared error (RMSE) of 0.28 for both the training and test datasets, while the Linear Regression approach resulted in an RMSE of 0.62 for both datasets. Further details on alternative feature selection methods can be found in Table 2. Utilizing feature ranking and importance scores facilitated the identification of the most pertinent features for our classification task. This feature selection process was reiterated on both imbalanced and balanced datasets, and the resulting feature set was employed for subsequent classification and model selection endeavors. Additionally, we conducted a correlation analysis to identify highly correlated features, focusing on those with correlations exceeding 0.8 and below 1.0.

**Table 2:** Selected features and corresponding feature selection methods.

Method	Parameters	Feature Count	Features
Random Forest Regressor	max$\_$depth = 10	15	’KURT’, ’MEAN$\_$RR’, ’HF$\_$PCT’, ’TP’, ’higuci’, ’pNN50’, ’VLF’, ’LF’, ’SDRR$\_$RMSSD$\_$REL$\_$RR’, ’SDRR$\_$REL$\_$RR’, ’SDRR$\_$RMSSD’, ’MEDIAN$\_$RR’, ’MEDIAN$\_$REL$\_$RR’, ’HR’, ’pNN25’
Lasso (L1)	$\alpha$ = 0.1	12	’MEAN$\_$RR’, ’MEDIAN$\_$RR’, ’pNN25’, ’SD2’, ’KURT’, ’KURT$\_$REL$\_$RR’, ’VLF’, ’LF’, ’LF$\_$NU’, ’HF’, ’HF$\_$NU’, ’LF$\_$HF’
Ridge (L2)	$\alpha$ = 0.1	5	’SDRR’, ’MEDIAN$\_$RR’, ’MEAN$\_$RR’, ’RMSSD’, ’SDSD’
SelectKBest	chi2 with 34 features	15	’MEAN$\_$RR’, ’MEDIAN$\_$RR’, ’HR’, ’pNN25’, ’SDSD’, ’SD1’, ’RMSSD’, ’LF’, ’SDRR$\_$RMSSD$\_$REL$\_$RR’, ’VLF’, ’VLF$\_$PCT’, ’LF$\_$PCT’, ’LF$\_$NU’, ’HF$\_$NU’, ’TP’
PCA	n$\_$components = 1 $\rightarrow$ 34	11	’TP’, ’VLF’, ’LF’, ’LF$\_$HF’, ’SD2’, ’MEAN$\_$RR’, ’SDRR’, ’MEDIAN$\_$RR’, ’VLF$\_$PCT’, ’HF’, ’pNN25’
Correlation	Spearman	15	’HR’, ’pNN50’, ’KURT’, ’SKEW’, ’MEAN$\_$REL$\_$RR’, ’MEDIAN$\_$REL$\_$RR’, ’SDRR$\_$RMSSD$\_$REL$\_$RR’, ’KURT$\_$REL$\_$RR’, ’SKEW$\_$REL$\_$RR’, ’VLF$\_$PCT’, ’LF$\_$PCT’, ’LF$\_$NU’, ’LF$\_$HF’, ’sampen’, ’higuci’
GA-1	n$\_$population=**5**, crossover$\_$proba=0.5, mutation$\_$proba=0.2, n$\_$generations=15, tournament$\_$size=3	8	’RMSSD’, ’SDRR$\_$RMSSD’, ’pNN25’, ’VLF’, ’LF’, ’HF$\_$PCT’, ’HF$\_$NU’, ’sampen’
GA-2	n$\_$population=**10**, crossover$\_$proba=0.5, mutation$\_$proba=0.2, n$\_$generations=15, tournament$\_$size=3	7	’MEDIAN$\_$RR’, ’SDRR$\_$RMSSD’, ’SD1’, ’SKEW’, ’VLF’, ’sampen’
GA-3	n$\_$population=**15**, crossover$\_$proba=0.5, mutation$\_$proba=0.2, n$\_$generations=15, tournament$\_$size=3	8	’MEDIAN$\_$RR’, ’HR’, ’pNN50’, ’SDSD$\_$REL$\_$RR’, ’SDRR$\_$RMSSD$\_$REL$\_$RR’, ’LF$\_$PCT’, ’HF’, ’higuci’
GA-4	n$\_$population=**20**, crossover$\_$proba=0.5, mutation$\_$proba=0.2, n$\_$generations=15, tournament$\_$size=3	10	’MEAN$\_$RR’, ’pNN25’, ’pNN50’, ’SD2’, ’MEDIAN$\_$REL$\_$RR’, ’RMSSD$\_$REL$\_$RR’, ’SKEW$\_$REL$\_$RR’, ’SDSD$\_$REL$\_$RR’, ’VLF$\_$PCT’, ’LF$\_$HF’

#### Semantic representation of knowledge

Since the feature selection methods showed similar results with the classification algorithm, we decided to organize this information in a simple, understandable way using an OWL ontology. This ontology categorizes different feature selection techniques and the features they select. This organized information can help us better understand and use the data. To create an HRV-based stress management system, we can use SPARQL, a well-known language for asking questions about data. SPARQL is important because it lets us ask complex questions, combine different datasets, and make sense of the data. It’s a useful tool for developers, researchers, and organizations looking to build smart, data-driven applications and solutions.

**Figure 3:**
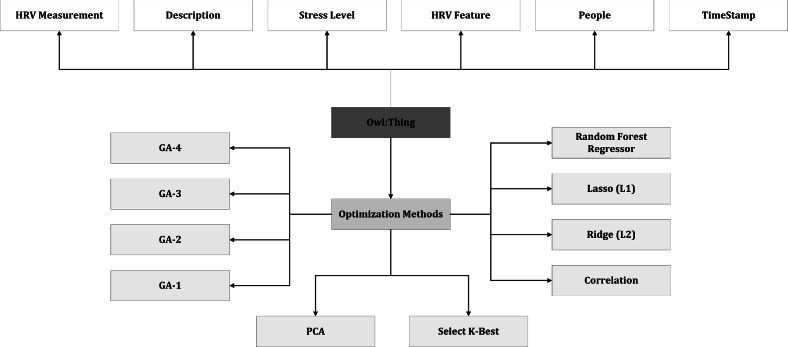
The high-level structure of our ontology.

In our designed and developed Ontology, the end-users can have the leverage to select a choice-based feature selection method and go for a stress-level determination. As the features are non-linearly dependent, we have achieved the best classification results with the genetic algorithm-based feature selection method. Some high-level examples of the sample SPARQL queries for stress management based on HRV features have been elaborated in Supplementary Material-5. In the examples of Supplementary Material-5, the “http://example.com/ontology/hrv$\#$” serves as the namespace for our ontology. Terms like “hasHRVFeature” and “hasValue” are used to describe relationships between objects, while terms like “HRVMeasurement”, “HRVFeature”, and “Person” classify different types of objects. We’ve visualized the overall structure of our ontology in Fig. 3 using OntoGraf. SPARQL, in this context, facilitates easy access to data, helps generate knowledge, and supports semantic reasoning. SPARQL queries are used to fetch information from RDF graphs. In RDF, information is represented in triples, each consisting of a subject, predicate, and object. The subject refers to the resource itself, the predicate indicates the property or connection of the resource, and the object is the value or another related resource. To ensure the correctness and coherence of our ontology, we’ve relied on the Hermit reasoner. It swiftly validates the consistency of our ontology and offers valuable insights into its logical structure in under a second. Thanks to its efficiency and compatibility with the OWL profile, Hermit has proven to be an ideal tool for our semantic ontology development.

#### Classification on imbalanced and balanced datasets

The classification outcomes were assessed based on HRV data, initially comprising 34 features without data augmentation. The original dataset yielded classification results and the Random Forest Classifier achieved the best accuracy score (accuracy of 97.9$\%$, alongside F1, Precision, Recall, and MCC scores all at 97.9$\%$ and an MCC of 96.3$\%$), detailed in Table 3. In subsequent experiments, balanced datasets were generated using SMOTE and ADASYN algorithms with 34 features for reclassification. Results are summarized in Tables 4 and 5. Across all scenarios, Random Forest Classifier consistently demonstrated superior performance compared to alternative classifiers, and on the balanced dataset achieved $\approx$ 2$\%$ more accuracy.

In this context of multi-class classification based on SWELL-KW dataset, using the best performing optimized Random Forest Classifier model, the utilization of 5-fold cross-validation facilitated a comprehensive assessment of the Random Forest Classifier model’s efficacy. A pivotal metric for evaluating the model’s consistency across varied SWELL-KW data subsets or iterations involved the computation of standard deviations for both training and testing curves. Predictive operations were initially conducted on the training and testing datasets using the Random Forest Classifier model, followed by the calculation of loss metrics, predominantly Mean Squared Error (MSE)^93^, for each balanced and imbalanced dataset. For individual iterations, the standard deviations of training and testing losses were determined as $\approx$ 0.162 and $\approx$ 0.140, and $\approx$ 0.175 and $\approx$ 0.158 for imbalanced and balanced datasets, respectively. To bolster the reliability of the standard deviation estimation, cross-validation was favored over a singular train-test division. The methodology encompassed repetitive implementations across multiple iterations (or 5-folds) within the cross-validation framework, enabling the accumulation of loss metrics for diverse data partitioning. Ultimately, the standard deviation of the used MSE loss metric was ascertained to assess the Random Forest Classifier model’s performance variability. Within the 5-fold cross-validation paradigm, the standard deviations for training and testing losses were found as $\approx$ 0.159 and $\approx$ 0.143, and $\approx$ 0.150 and $\approx$ 0.140 for imbalanced and balanced datasets, respectively. A higher standard deviation in test losses compared to training losses typically suggests potential overfitting or model inconsistency. In the present study, such disparity was not observed. Consequently, consistent standard deviations across both training and testing losses affirm the Random Forest model’s stable performance in multi-class classification utilizing SWELL-KW HRV data.

To optimize the feature space and address potential biases, we conducted feature engineering on the 34 features, subsequently applying stress-level classification. The classification analysis revealed superior performance with the GA-3 and GA-4 feature selection methods, as detailed in Table 2. Further classification outcomes are provided in Tables6 and 7. Notably, the Random Forest Classifier demonstrated exceptional performance, achieving an accuracy score of 99.4$\%$ (F1: 99.4$\%$, Precision: 99.4$\%$, Recall: 99.4$\%$, MCC: 98.8$\%$). To ensure robustness, we conducted additional experiments using balanced datasets generated by the SMOTE (Tables8 and 9) and ADASYN (Table srefclassifysps6 and 11) algorithms. Remarkably, the Random Forest Classifier consistently outperformed other classifiers, achieving an accuracy score of 99.8$\%$ (F1: 99.8$\%$, Precision: 99.8$\%$, Recall: 99.8$\%$, MCC: 99.1$\%$) across all datasets. This suggests that both data balancing methods had a similar positive impact on the original HRV dataset. Comparatively, all other feature selection methods, when combined with RF classification, yielded lower accuracy scores than the GA-3 and GA-4 methods. For a comprehensive overview of the genetic algorithm-based feature selection process followed by random forest classification (see Algorithm 9).

Based on detailed experiments, both augmented (balanced) and unaugmented (imbalanced) tabular data yield a competitive classification accuracy. This suggests that the original dataset and model are performing near optimally under the given conditions. However, data augmentation improved classification metrics by providing more varied training examples and potentially enhancing generalization. Random Forest Classification models are inherently robust to data variations. Consequently, if a model is highly effective at generalizing from the available data without overfitting, it may perform similarly well on both augmented and unaugmented datasets, which is observed in this scenario with the Random Forest Classification model.

**Table 3:** Classification of original HRV data with 34 features without data augmentation (D1).

Models	Precision	Recall	F1-score	Mean accuracy	MCC	Standard deviation of Accuracies ($\%$)
LR	59.0$\%$	59.0$\%$	59.0$\%$	59.0$\%$	0.530	0.164
LDA	61.0$\%$	61.0$\%$	61.0$\%$	61.0$\%$	0.551	0.152
KNN	97.0$\%$	97.0$\%$	97.0$\%$	97.0$\%$	0.890	0.002
NB	48.0$\%$	48.0$\%$	48.0$\%$	48.0$\%$	0.411	0.129
DT	97.0$\%$	97.0$\%$	97.3$\%$	97.0$\%$	0.900	0.010
Bagging	97.5$\%$	97.5$\%$	97.5$\%$	97.5$\%$	0.910	0.002
Ada Boost	65.0$\%$	65.5$\%$	65.5$\%$	65.5$\%$	0.590	0.972
Grad Boost	88.0$\%$	88.0$\%$	88.5$\%$	88.5$\%$	0.810	0.241
RF	97.9$\%$	97.9$\%$	97.9$\%$	97.9$\%$	0.918	0.000

**Table 4:** Classification of SMOTE-generated augmented HRV data with 34 features (D2).

Models	Precision	Recall	F1-score	Mean accuracy	MCC	Standard deviation of Accuracies ($\%$)
LR	63.0$\%$	63.0$\%$	63.0$\%$	59.0$\%$	0.545	0.099
LDA	64.0$\%$	64.0$\%$	64.0$\%$	64.0$\%$	0.583	0.116
KNN	99.0$\%$	99.0$\%$	99.0$\%$	99.0$\%$	0.905	0.002
NB	51.0$\%$	51.0$\%$	51.0$\%$	51.0$\%$	0.475	0.164
DT	99.2$\%$	99.0$\%$	99.3$\%$	99.0$\%$	0.911	0.013
Bagging	99.5$\%$	99.5$\%$	99.5$\%$	99.5$\%$	0.918	0.005
Ada Boost	69.0$\%$	69.5$\%$	69.5$\%$	69.5$\%$	0.625	0.971
Grad Boost	91.0$\%$	92.0$\%$	91.0$\%$	91.0$\%$	0.862	0.241
RF	99.9$\%$	99.9$\%$	99.9$\%$	99.9$\%$	0.927	0.000

From the resultant classification performance tables, accuracy measures the proportion of correct predictions, while F1-score represents the harmonic mean of precision and recall. MCC balances the true positives, true negatives, false positives, and false negatives. These metrics play a pivotal role in assessing model performance. By monitoring these metrics across both training and test datasets, we can gauge the used ML model’s generalization capabilities. Stratification ensures that each fold of the cross-validation retains the same proportion of classes as the complete dataset, making it particularly effective for imbalanced datasets. Consistency between these metrics, when coupled with stratified cross-validation, indicates that the model is likely not overfitting, ensuring it performs well on unseen data and across balanced and imbalanced datasets.

Techniques, such as K-fold cross-validation with stratification, provide a robust estimate of the ML model’s performance by training and validating across multiple subsets of the data, ensuring a balanced representation of classes in each fold. Data balancing methods, such as over-sampling the minority class or under-sampling the majority class, combined with stratification, ensure the model learns patterns from both classes equally. Meanwhile, hyperparameter tuning fine-tunes the model’s complexity. Collectively, these strategies, especially when stratified techniques are employed, ensure the Random Forest classification model is well-optimized, trained on imbalanced and balanced datasets, and tested robustly. This significantly reduces the risk of overfitting, enhancing its reliability and performance in predictive analysis tasks.

**Table 5:** Classification of ADASYN-generated augmented HRV data with 34 features (D3).

Models	Precision	Recall	F1-score	Mean accuracy	MCC	Standard deviation of Accuracies ($\%$)
LR	63.0$\%$	63.0$\%$	63.0$\%$	63.0$\%$	0.550	0.360
LDA	63.8$\%$	63.8$\%$	63.8$\%$	63.8$\%$	0.585	0.152
KNN	99.0$\%$	99.0$\%$	99.0$\%$	99.0$\%$	0.901	0.033
NB	51.0$\%$	51.0$\%$	51.0$\%$	51.0$\%$	0.480	0.117
DT	99.2$\%$	99.0$\%$	99.3$\%$	99.0$\%$	0.908	0.008
Bagging	99.2$\%$	99.2$\%$	99.2$\%$	99.2$\%$	0.915	0.002
Ada Boost	69.4$\%$	69.4$\%$	69.4$\%$	69.4$\%$	0.632	0.327
Grad Boost	91.5$\%$	91.5$\%$	91.5$\%$	91.5$\%$	0.869	0.189
RF	99.9$\%$	99.9$\%$	99.9$\%$	99.9$\%$	0.930	0.000

**Table 6:** Classification of optimized original HRV data (D1) with 8 features (GA-3).

Models	Precision	Recall	F1-score	Mean accuracy	MCC	Standard deviation of Accuracies ($\%$)
LR	59.8$\%$	59.0$\%$	59.0$\%$	60.0$\%$	0.520	0.477
LDA	59.5$\%$	59.0$\%$	59.0$\%$	59.5$\%$	0.510	0.103
KNN	98.2$\%$	98.2$\%$	98.0$\%$	98.2$\%$	0.890	0.011
NB	59.3$\%$	59.0$\%$	59.0$\%$	59.0$\%$	0.500	0.163
DT	98.0$\%$	98.0$\%$	98.0$\%$	98.0$\%$	0.882	0.008
Bagging	98.2$\%$	98.2$\%$	98.1$\%$	98.2$\%$	0.889	0.006
Ada Boost	66.0$\%$	66.0$\%$	66.0$\%$	66.0$\%$	0.594	0.061
Grad Boost	87.0$\%$	87.5$\%$	87.5$\%$	87.5$\%$	0.814	0.214
RF	98.4$\%$	98.4$\%$	98.4$\%$	98.4$\%$	0.902	0.000

**Table 7:** Classification of optimized original HRV data (D1) with 10 features (GA-4).

Models	Precision	Recall	F1-score	Mean accuracy	MCC	Standard deviation of Accuracies ($\%$)
LR	60.0$\%$	61.0$\%$	61.0$\%$	61.0$\%$	0.525	0.126
LDA	59.0$\%$	59.0$\%$	59.0$\%$	59.0$\%$	0.505	0.149
KNN	98.1$\%$	98.1$\%$	98.1$\%$	98.1$\%$	0.887	0.036
NB	54.0$\%$	54.0$\%$	54.0$\%$	54.0$\%$	0.475	0.161
DT	98.0$\%$	98.0$\%$	98.0$\%$	98.0$\%$	0.880	0.010
Bagging	98.0$\%$	98.0$\%$	98.0$\%$	98.0$\%$	0.887	0.014
Ada Boost	66.0$\%$	66.5$\%$	66.5$\%$	66.5$\%$	0.586	0.621
Grad Boost	87.0$\%$	87.0$\%$	87.5$\%$	87.5$\%$	0.820	0.209
RF	98.4$\%$	98.4$\%$	98.4$\%$	98.4$\%$	0.908	0.000

**Table 8:** Classification of optimized SMOTE HRV data (D2) with 8 features (GA-3).

Models	Precision	Recall	F1-score	Mean accuracy	MCC	Standard deviation of Accuracies ($\%$)
LR	61.5$\%$	61.5$\%$	61.0$\%$	61.0$\%$	0.530	0.165
LDA	64.5$\%$	64.5$\%$	64.0$\%$	64.0$\%$	0.558	0.152
KNN	99.1$\%$	99.2$\%$	99.0$\%$	99.0$\%$	0.894	0.001
NB	51.0$\%$	51.5$\%$	51.5$\%$	51.5$\%$	0.442	0.129
DT	99.0$\%$	99.1$\%$	99.1$\%$	99.0$\%$	0.920	0.011
Bagging	99.5$\%$	99.5$\%$	99.5$\%$	99.5$\%$	0.926	0.003
Ada Boost	69.0$\%$	69.5$\%$	69.5$\%$	69.5$\%$	0.610	0.973
Grad Boost	91.5$\%$	92.0$\%$	91.5$\%$	91.5$\%$	0.836	0.232
RF	99.8$\%$	99.6$\%$	99.8$\%$	99.8$\%$	0.930	0.000

**Table 9:** Classification of optimized SMOTE HRV data (D2) with 10 features (GA-4).

Models	Precision	Recall	F1-score	Mean accuracy	MCC	Standard deviation of Accuracies ($\%$)
LR	61.0$\%$	61.0$\%$	61.0$\%$	61.0$\%$	0.505	0.165
LDA	64.0$\%$	64.5$\%$	64.0$\%$	64.0$\%$	0.528	0.152
KNN	99.0$\%$	99.2$\%$	99.0$\%$	99.0$\%$	0.910	0.002
NB	51.5$\%$	51.5$\%$	51.5$\%$	51.5$\%$	0.419	0.129
DT	99.1$\%$	99.1$\%$	99.1$\%$	99.0$\%$	0.914	0.011
Bagging	99.0$\%$	99.5$\%$	99.5$\%$	99.5$\%$	0.919	0.003
Ada Boost	69.5$\%$	69.5$\%$	69.5$\%$	69.5$\%$	0.617	0.973
Grad Boost	92.0$\%$	92.0$\%$	91.5$\%$	91.5$\%$	0.826	0.231
RF	99.7$\%$	99.9$\%$	99.8$\%$	99.8$\%$	0.917	0.000

**Table 10:** Classification of optimized ADASYN HRV data (D3) with 8 features (GA-3).

Models	Precision	Recall	F1-score	Mean accuracy	MCC	Standard deviation of Accuracies ($\%$)
LR	62.0$\%$	63.5$\%$	63.0$\%$	63.0$\%$	0.509	0.164
LDA	63.5$\%$	64.0$\%$	64.0$\%$	64.0$\%$	0.531	0.151
KNN	99.0$\%$	99.0$\%$	99.0$\%$	99.0$\%$	0.890	0.002
NB	52.0$\%$	52.0$\%$	52.0$\%$	52.0$\%$	0.431	0.128
DT	99.0$\%$	99.0$\%$	99.0$\%$	99.0$\%$	0.898	0.011
Bagging	99.5$\%$	99.4$\%$	99.5$\%$	99.4$\%$	91.1	0.001
Ada Boost	69.0$\%$	69.5$\%$	69.5$\%$	69.5$\%$	59.6	0.973
Grad Boost	91.5$\%$	92.0$\%$	91.5$\%$	92.0$\%$	0.805	0.251
RF	99.7$\%$	99.8$\%$	99.8$\%$	99.8$\%$	0.919	0.000

**Table 11:** Classification of optimized ADASYN HRV data (D3) with 10 features (GA-4).

Models	Precision	Recall	F1-score	Mean accuracy	MCC	Standard deviation of Accuracies ($\%$)
LR	62.0$\%$	63.0$\%$	63.0$\%$	63.0$\%$	0.506	0.164
LDA	63.0$\%$	64.0$\%$	64.0$\%$	64.0$\%$	0.536	0.152
KNN	99.1$\%$	99.1$\%$	99.1$\%$	99.1$\%$	0.896	0.003
NB	52.0$\%$	52.0$\%$	52.0$\%$	52.0$\%$	0.439	0.129
DT	99.1$\%$	99.0$\%$	99.1$\%$	99.0$\%$	0.901	0.010
Bagging	99.4$\%$	99.4$\%$	99.4$\%$	99.4$\%$	0.906	0.001
Ada Boost	70.0$\%$	69.5$\%$	69.5$\%$	69.5$\%$	0.578	0.972
Grad Boost	91.5$\%$	92.0$\%$	91.5$\%$	92.0$\%$	0.812	0.241
RF	99.8$\%$	99.8$\%$	99.8$\%$	99.8$\%$	0.922	0.000

**Algorithm 9:**
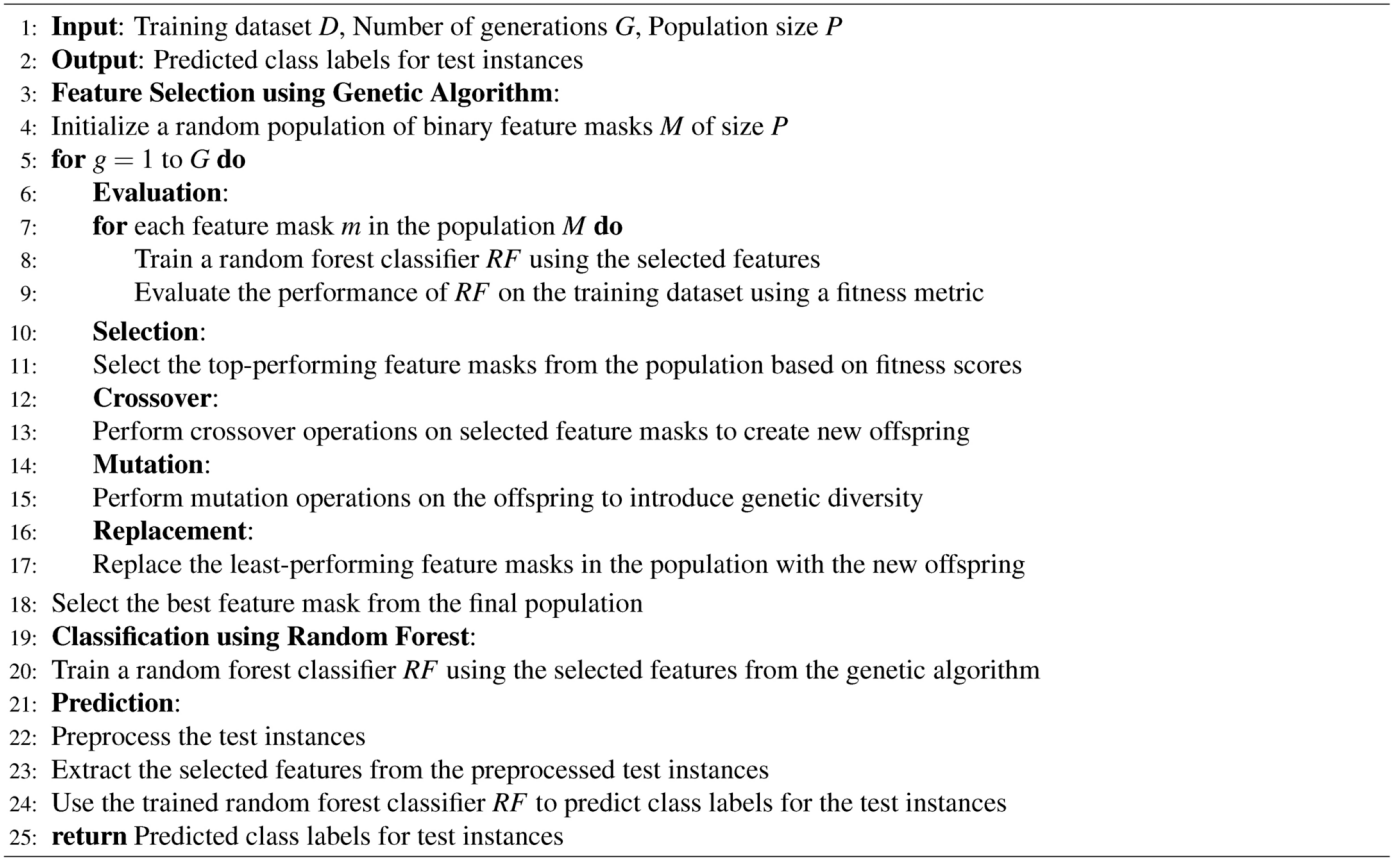
Genetic Algorithm-based Feature Selection and Random Forest Classification

**Table 12:** The summary of hyperparameter optimization on imbalanced and balanced datasets.

Combination	Data Type	Parameters
RF	Imbalanced (D1)	’criterion’: ’entropy’, ’max$\_$depth’: 6, ’n$\_$estimators’: 200
GA-3 + RF	Imbalanced (D1)	’criterion’: ’entropy’, ’max$\_$depth’: 8, ’n$\_$estimators’: 300
GA-4 + RF	Imbalanced (D1)	’criterion’: ’entropy’, ’max$\_$depth’: 8, ’n$\_$estimators’: 300
GA-3 + RF	Balanced (SMOTE) (D2)	’criterion’: ’entropy’, ’max$\_$depth’: 6, ’n$\_$estimators’: 200
GA-4 + RF	Balanced (SMOTE) (D2)	’criterion’: ’entropy’, ’max$\_$depth’: 6, ’n$\_$estimators’: 200
GA-3 + RF	Balanced (ADASYN) (D3)	’criterion’: ’entropy’, ’max$\_$depth’: 6, ’n$\_$estimators’: 200
GA-4 + RF	Balanced (ADASYN) (D3)	’criterion’: ’entropy’, ’max$\_$depth’: 6, ’n$\_$estimators’: 200

As an example, for Table 11, the MCC calculations were validated against the provided classifier performance metrics. The dataset contained 666,720 rows, evenly distributed across three classes, with each class containing 222,240 samples. For Table 11, the Random Forest classifier achieved an accuracy of 99.8% and an MCC of 0.922. Assuming a high-performance scenario, the confusion matrix values were derived as follows: True Positives (TP) = 221,795, True Negatives (TN) = 443,590, False Positives (FP) = 445, and False Negatives (FN) = 445. Substituting these values into the MCC formula, we obtain-

6 $$\begin{aligned} MCC = \frac{(221,795 \cdot 443,590 - 445 \cdot 445)}{\sqrt{(221,795 + 445)(221,795 + 445)(443,590 + 445)(443,590 + 445)}} \end{aligned}$$

resulting in

7 $$\begin{aligned} MCC = \frac{98,362,470,250 - 198,025}{\sqrt{49,408,017,600,000}} \end{aligned}$$

producing

8 $$\begin{aligned} MCC \approx 0.922. \end{aligned}$$

The same process has been carried out for other tables to calculate the MCC value.

## Discussion

### Experimental findings

The analysis presents a comprehensive exploration of data balancing, feature selection, and semantic representation techniques in the context of stress management using HRV data. Initially, the study addresses the issue of data imbalance within the SWELL-KW dataset, employing SMOTE and ADASYN algorithms to augment the data. Both methods substantially increase the dataset size while maintaining data privacy, with ADASYN, the more advanced version, producing slightly more augmented data. Feature selection is then conducted using various techniques, including Linear Regression, Random Forest Regressor, SelectKBest, PCA, Lasso, Ridge, and Genetic Algorithms (GA). The study compares these methods and opts for Random Forest Regressor due to its superior performance, particularly in reducing the root-mean-squared error (RMSE) for both training and test datasets. The selected features are employed for subsequent classification tasks, with Random Forest consistently outperforming other classifiers across all scenarios.

The study further explores semantic representation using OWL ontology to organize feature selection information in a structured and understandable manner. SPARQL is highlighted as a valuable tool for querying and making sense of data, particularly in the context of building HRV-based stress management systems. Classification on both imbalanced and balanced datasets demonstrates the effectiveness of the Random Forest Classifier, particularly when combined with feature selection methods such as Genetic Algorithms (GA-3 and GA-4). The study emphasizes the importance of feature engineering in optimizing classification performance, with Random Forest consistently yielding high accuracy scores across all datasets.

Grid search and cross-validation have emerged as vital techniques in stress level classification using HRV data. Grid search facilitates the discovery of optimal hyperparameter values, while cross-validation offers a reliable estimate of model performance, aiding in evaluation, selection, and hyperparameter optimization. Together, these approaches enhance the creation of more effective machine-learning models. The SWELL-KW dataset used in this study is well-organized, resulting in consistent classification outcomes for both balanced and imbalanced datasets. Additionally, calibrated classification and stratification techniques have played crucial roles in verifying model performance, improving generalization, and fostering trust in predictive models. By ensuring accurate probabilities and representative data sampling, these practices contribute to more informed decision-making and more reliable predictive insights.

Utilizing an ontology for knowledge representation in feature and stress management offers a standardized and structured approach, enhancing the acquisition and interpretation of stress-related concepts. This approach fosters effective decision-making, personalized treatments, and integration of diverse knowledge sources. Ontologies improve querying and reasoning processes, leading to more accurate information retrieval and extraction of meaningful results. In stress management, ontologies form the basis for encoding domain knowledge, enable rules-based approaches, and facilitate personalization and adaptation of management strategies. Overall, the analysis provides valuable insights into the process of data balancing, feature selection, semantic representation, and classification in stress management using HRV data. The study’s rigorous methodology and comprehensive evaluation of techniques contribute to the advancement of knowledge in this field, offering potential avenues for further research and development of effective stress management systems.

### Metric convergence at high performance levels

When classification models achieve high accuracy, the values of other metrics such as precision, recall, F1 score, and MCC tend to converge. This convergence occurs because at high levels of performance, errors (false positives and false negatives) are minimal, making all metrics approach their ideal values (close to 1). In Table 11 , the random forest model shows an average accuracy of 99.8%, with precision, recall, and F1 scores also at 99.8%. Similarly, MCC reaches 0.922, reflecting a strong correlation between predictions and actual labels. Such consistency of metrics is expected when performance is close to perfect, confirming the robustness of the model.

MCC considers all elements of the confusion matrix to evaluate classification performance, which makes it particularly valuable for imbalanced datasets. It considers various types of errors, has a specific interpretive range, accounts for negative instances, and aids in model selection and comparison. Unlike accuracy, which can be misleading in this context, MCC provides a balanced measure of the correlation between predicted and actual labels, ensuring reliability even under different class distributions. The results in the Tables 3, 4, 5, 6, 7, 8, 9, 10, 11 show that for less effective models (e.g., LR, LDA, NB, AdaBoost), the MCC values are always lower than the accuracy, while for high-accuracy models (e.g., KNN, DT, Bagging, RF), the MCC values closely match the accuracy, confirming the importance of its accuracy in the comprehensive evaluation of classifier performance (Table 12).

Due to well-calibrated decision boundaries, KNN, DT, Bagging, and RF models closely align MCC and accuracy values, ensuring that predictions are robust across classes and not biased by class imbalance. In this context, the results confirm the reliability, fairness, and usefulness of the models in real-world applications on balanced or imbalanced datasets. Compared to models operating in the 50-70% accuracy range, where accuracy may overestimate performance due to dominant class predictions, the reported models exhibit a tight coupling between MCC and accuracy metrics^94–96^. This coupling indicates superior performance due to proper handling of class imbalance and minimal errors. For example, the precision and recall values of random forests confirm their ability to accurately capture true positives while minimizing false negatives and false positives. This suggests that MCC is a more reliable measure than accuracy alone, and it ensures fairness and robustness of classification assessment, especially in critical applications such as HRV-based stress management.

### Classification explanation with tree-explainer

Tree interpretations are crucial for understanding machine learning predictions, as they offer interpretable and transparent insights into the decision-making process of tree-based models. In the context of the increasing demand for transparency and explainability in AI, tree interpretation remains pivotal in constructing trustworthy and ethical AI systems. SHAP stands out as a significant tree-based explainer due to its interpretability, versatility, and ability to provide valuable insights into the decision-making process of tree-based machine learning models, such as Random Forest. Utilizing different color codes, SHAP denotes the importance of attributes, facilitating the interpretation of results. In our study, we employed SHAP for Random Forest classification, with the algorithm outlined in Algorithm 10.

**Algorithm 10:**
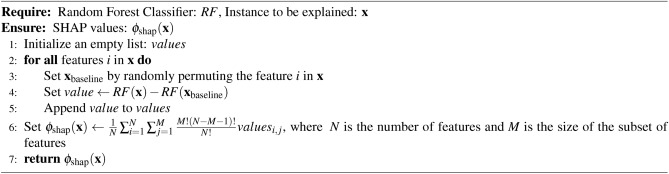
SHAP for Random Forest Classification as a Tree-Explainer

The optimization process employing a Genetic Algorithm (GA) significantly enhanced the effectiveness and efficiency of Machine Learning (ML) classification models by refining the HRV feature set. Through the GA’s capacity to eliminate irrelevant and redundant features, the focus was directed towards the most informative attributes. Results demonstrated that the GA in conjunction with the Random Forest (RF) algorithm achieved the highest accuracy when employing eight features (GA-3 method) and ten features (GA-4 method), as detailed in Table 2. Moreover, the RF classification performed similarly with ten features (GA-4 method). Notably, this study marks the pioneering application of a meta-heuristic optimization algorithm for feature engineering in the SWELL-KW dataset, attributed to the dataset’s inherent non-linear feature relationships. Nonetheless, it’s essential to acknowledge that the resultant classification, based on these refined attributes, may appear as a black box to non-experts. Therefore, ensuring the accuracy and interpretability of the model remains paramount. To facilitate comprehension, Algorithm 10 elucidates the RF classification outcomes utilizing SHAP with eight features. This analysis considers the top features selected by GA for each predicted class - class ’0’, class ’1’, and class ’2’, corresponding to interruption, no stress, and time pressure, respectively.

**Figure 4:**
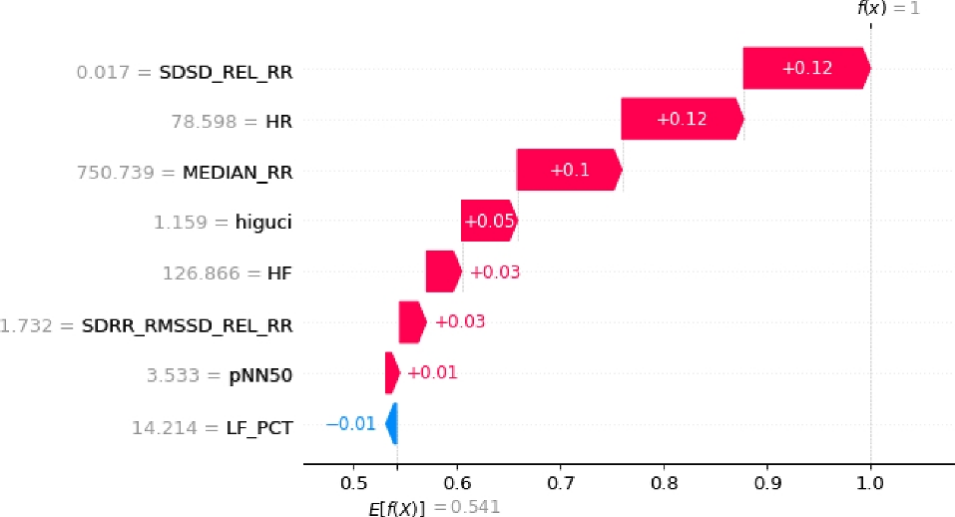
The explanation towards the feature distribution to classify class ’0’.

**Figure 5:**
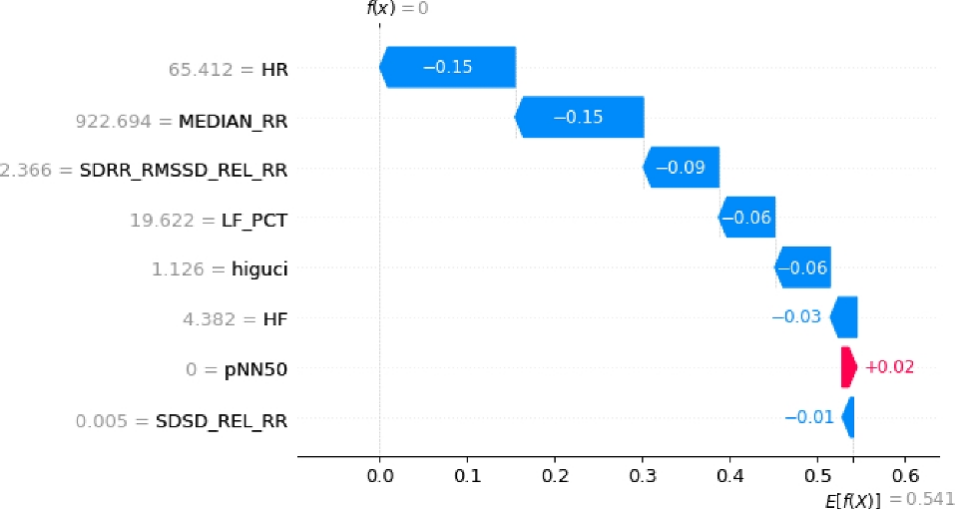
The explanation towards the feature distribution to classify class ’1’.

**Figure 6:**
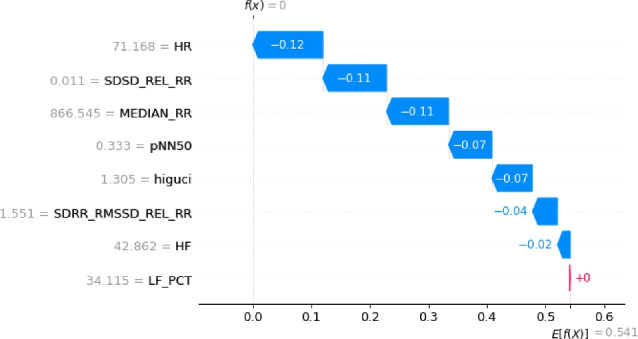
The explanation towards the feature distribution to classify class ’2’.

The results of this study are visualized in Figs. 4, 5, and 6. Features of greater importance are depicted with warmer colors, such as red, while those of lesser importance are represented with cooler colors, like blue. This color scheme in the SHAP graphs aids in providing a clear and visually engaging interpretation of feature contributions, enhancing comprehension of our Random Forest Classification model’s predictions.

Moreover, the SHAP violin plots are a very useful way to visualize the distribution of the SHAP values for each feature across different instances in the used HRV dataset.

**Figure 7:**
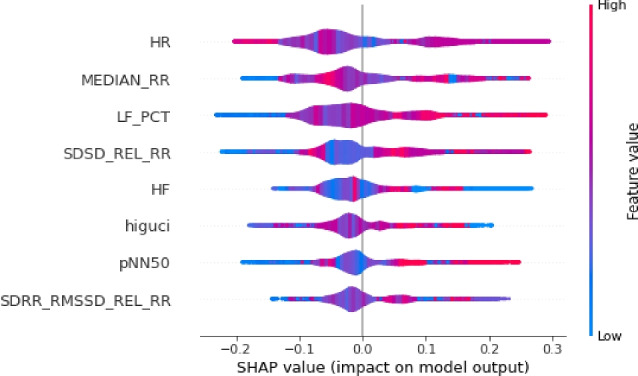
The explanation towards the feature distribution to classify class ’0’.

**Figure 8:**
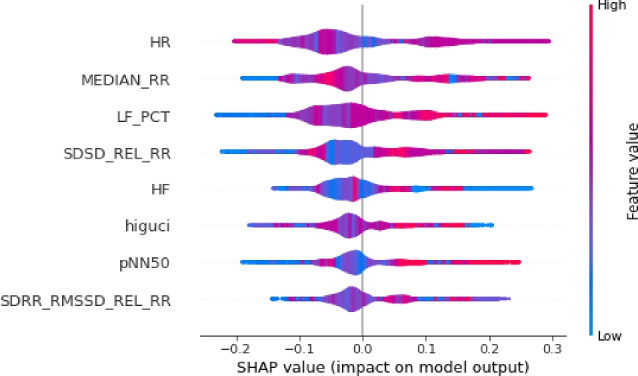
The explanation towards the feature distribution to classify class ’1’.

**Figure 9:**
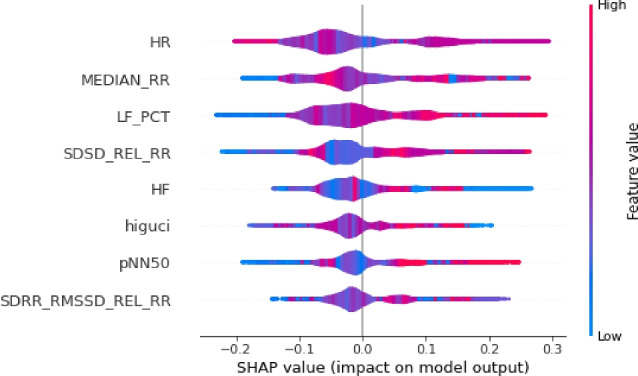
The explanation towards the feature distribution to classify class ’2’.

In our study, we utilized SHAP violin plots (Figs. 7, 8, and 9) to visually assess the distribution of SHAP values across selected features within three distinct target classes. This approach enabled a comparative analysis of how each feature influences the predictions of the Random Forest Classification model across different groups. While bar charts in SHAP concentrate on individual predictions, the implementation of violin plots offers a broader perspective by showcasing the distribution of feature importance across multiple predictions. This holistic visualization aids in the identification of overarching patterns and trends in the model’s behavior.

### Ethical aspects of AI

In this research, the ethical aspects of AI have been explained in Table 13.

**Table 13:** The adopted approaches for attaining ethical aspects of AI in this research problem.

Ethical AI considerations	Adopted approaches
Collection of ethical data	We have used a publicly available anonymous HRV dataset for the experiment.
Test for bias	The use of calibrated classification, stratification, grid search, and metrics, such as MCC, to test for bias in our models.
Test for fairness	The use of SMOTE and ADASYN algorithms for data balancing and a comparative analysis between balanced and imbalanced datasets.
Model Explainability	The use of ML-based local model Explanation with Tree-based approach (e.g., SHAP).
Ethical principles	The testing for bias, fairness, model scalability, and explainability
Privacy consideration	Data balancing with sampling algorithms, such as SMOTE and ADASYN preserve data privacy.
Iterate and improve	Incremental modeling for handling growing HRV data, continuous training-validation-testing, and automatic stress detection.

### Comparison with previous studies

In the realm of stress level classification, various studies have leveraged diverse datasets, feature selection techniques, and machine learning algorithms. Performance metrics such as accuracy, MCC, F1-score, recall, and precision have been used to evaluate the efficacy of these approaches. Notably, we achieved the highest accuracy rates in the (GA + RF) machine learning pipeline on the balanced and imbalanced SWELL-KW dataset, while Mortensen et al. demonstrated good accuracy scores with a 1D-CNN classifier on the same dataset with more features. Moreover, Ghose et al. achieved a very good accuracy score with three features and K-NN model; however, like other studies, they have not verified model fairness and biases. Additionally, feature reduction methods, such as genetic algorithm-based selection, have shown promise in enhancing classification accuracy, as evidenced by the findings of our study. The comparative results have been captured in the Supplementary Material-6.

### Limitations and future scope

GA-based searching and SHAP-based searching are fundamentally different approaches with distinct purposes and applications, leading to different rankings of HRV features for selection in the optimal feature set. GA-based searching methods are ideal for global optimization problems, focusing on finding the best solution within a large search space. Conversely, SHAP-based model agnostic searching emphasizes both global and local interpretability and explanation of black-box models, offering insights into overall model predictions. The choice between these approaches hinges on the analysis’s specific goal, whether it prioritizes global optimization or local interpretability and explanation. Furthermore, this study maintains fixed parameters such as n$\_$population and others (e.g., crossover$\_$proba=0.5, mutation$\_$proba=0.2, n$\_$generations=15, tournament$\_$size=3) in the GA. Future investigations may delve into varying these fixed components and employing alternative meta-heuristic algorithms for further analysis.

In HRV-based stress management, an ontology serves to represent domain-specific knowledge, elucidating relationships, concepts, and properties pertinent to stress and its management. This study provides a simplified case, with the actual ontology design contingent upon specific requirements and context. The ontology’s scope can expand to encompass additional stressors, coping mechanisms, procedures, and relevant concepts. Integration with external knowledge sources or ontologies can augment its capacity, enriching knowledge representation with classes, properties, hierarchies, relationships, rules, inference, and annotations. Rules within the ontology can infer relationships or provide recommendations based on acquired knowledge. Metadata, including definitions, synonyms, or references to external resources, can enhance ontology elements. The effectiveness of ontology-based querying, reasoning, and rule-based systems relies on the ontology’s design quality and completeness, as well as the availability of pertinent data and suitable inferences.

## Conclusions

The HRV serves as an indicator of the equilibrium between the sympathetic and parasympathetic nervous systems. Elevated stress levels often result in diminished HRV, indicating an imbalance in the autonomic nervous system. This reduced HRV is closely linked to various mental health difficulties, such as anxiety and depression. Prolonged exposure to stress can perpetuate low HRV, adversely impacting mental wellness. However, techniques like HRV biofeedback and stress management strategies show promise in enhancing both HRV and mental health outcomes. In a comprehensive three-part study, the first phase outlines a machine learning pipeline aimed at optimizing and categorizing HRV datasets into distinct class labels. This involves employing feature selection methods like Genetic Algorithms in conjunction with machine learning classifiers such as Random Forests. The second phase focuses on addressing and refining model biases and fairness by employing techniques like SMOTE and ADASYN to balance the dataset. The study then repeats the experimentation process using these balanced datasets. The resulting classification outcomes demonstrate superior performance compared to existing methods, both on feature-optimized balanced and imbalanced datasets. In the third phase, a semantic ontology is devised to represent the optimized features and acquired knowledge, facilitating effective reasoning and querying. This ontology lays the groundwork for designing a rule-based stress management system. Additionally, the study demonstrates the interpretability of the model by employing the SHAP tree-based explainer, ensuring the ethical effectiveness and trustworthiness of the classification model. Furthermore, integrating feature optimization and AI-driven predictive analysis into an ontology-based stress management approach can harness the potential of HRV data for stress assessment, personalized intervention, early detection, and overall well-being. Leveraging physiological data alongside domain expertise empowers individuals to proactively address stress and enhance their overall health.

## Supplementary Information

Supplementary Information 1.

Supplementary Information 2.

Supplementary Information 3.

Supplementary Information 4.

Supplementary Information 5.

Supplementary Information 6.

## Data Availability

The used HRV dataset is publicly available at https://www.kaggle.com/datasets/qiriro/swell-heart-rate-variability-hrv (accessed: 20.06.2023) and http://cs.ru.nl/~skoldijk/SWELL-KW/Dataset.html (accessed: 20.06.2023). The codes are available at https://github.com/ayan1c2/HRV$\_$Swell.git.

## Abbreviations

HRV: Heart rate variability
ECG: Electrocardiography
PPG: Photo-plethysmography
X-AI: Explainable AI
SWELL-KW: SWELL knowledge work
SMOTE: Synthetic minority oversampling technique
ADASYN: Adaptive synthetic
ML: Machine learning
MCC: Mathew’s coefficient
SHAP: Shapley additive explanations
PCA: Principal component analysis
GA: Genetic algorithm
RF: Random forest
LDA: Linear discriminant analysis
DT: Decision tree
SVC: Support vector classifier
NB: Naive Bayes
LR: Logistic regression
KNN: K-nearest neighbor
GB: Gradient boosting
MCC: Mathew’s coefficient
WHO: World Health Organization
AI: Artificial intelligence
GDPR: General data protection regulation
CPU: Central processing unit
RDF: Resource description framework
OWL: Web Ontology Language
SPARQL: SPARQL Protocol and RDF Query Language
