# Supplementary material for: Stress management with HRV following AI, semantic ontology, genetic algorithm and tree explainer
Source: Sci Rep. 2025 Feb 17;15:5755. doi: 10.1038/s41598-025-87510-w (PMC11833117; doi:10.1038/s41598-025-87510-w)
Supplement: Supplementary file 1 — Supplementary Information 1. [file 41598_2025_87510_MOESM1_ESM.docx]

**Supplementary Table 1.** A qualitative comparison table showcasing the novel contributions of our study compared to existing research.

| **Aspect** | **Existing Studies** | **Our Novel Contribution** |
| --- | --- | --- |
| Feature Selection Techniques | Various studies have employed standard feature selection techniques followed by machine learning models. For example, Muhajir et al. achieved 70% accuracy on linear analysis and 60% accuracy on non-linear analysis using sixteen HRV features. Giannakakis et al. utilized sixteen features with an SVM classifier and achieved 84.4% accuracy with transformed features. | Utilization of meta-heuristic algorithms for feature optimization to create a relevant feature set, particularly for non-linear features. |
| Data Balancing Techniques | No studies have focused on data balancing techniques to address imbalanced datasets. Bobade et al. conducted binary and multi-class classification on the WESAD dataset using machine learning classifiers, obtaining accuracy scores of 84.32% and 95.21% respectively with seven features. | Implementation of standard oversampling techniques like SMOTE and ADASYN to balance data and conducting classification on both imbalanced and balanced datasets to ensure model fairness. |
| Interpretability of Classification | A few studies have explored methods to explain classification outcomes. For example, Ghose et al. achieved a 99.3% accuracy score with three features using the k-nearest neighbors (k-NN) algorithm. | Explanation of classification outcomes using tree explainer methods such as SHAP. |
| Ethical Considerations | Limited studies have addressed ethical considerations in AI applications. | Attention to ethical aspects of AI applications. |
| Knowledge Representation | There is a lack of emphasis on semantic representation for efficient data and knowledge management in existing studies. | Implementation of semantic representation to efficiently manage both data and knowledge derived from the study. |
