# Supplementary material for: Stress management with HRV following AI, semantic ontology, genetic algorithm and tree explainer
Source: Sci Rep. 2025 Feb 17;15:5755. doi: 10.1038/s41598-025-87510-w (PMC11833117; doi:10.1038/s41598-025-87510-w)
Supplement: Supplementary file 2 — Supplementary Information 2. [file 41598_2025_87510_MOESM2_ESM.docx]

**Supplementary Table 2.** Different feature engineering Methods in context.

| **Method Name** | **Description** |
| --- | --- |
| Filter Methods | Filter methods assess the importance of features by looking at their statistical properties alone in the learning process. Common methods include correlation-based approaches (e.g., the Pearson coefficient of correlation), information sharing, chi-square testing, and thresholds of variance. These approaches assess or rank features by their individual properties, and then choose the highest-ranked features.  The correlation coefficient measures the linear relationship between a feature and the target variable. Features with high absolute correlation coefficients (close to 1 or -1) are considered highly correlated with the target variable and may be good candidates for feature selection. |
| Wrapper Methods | Wrapper methods assess the effectiveness of a feature subset by evaluating the learning algorithm on different subsets of features. It necessitates choosing a subset of attributes, training the model, and evaluating its effectiveness. Recursive Feature Elimination (RFE) is a popular method of wrapper design that initially executes all features and then iteratively disregards the least significant features based on the model's effectiveness. |
| Embedded Methods | Embedded methods take part in the learning process itself, during which feature selection is incorporated into the learning algorithm. These approaches are intended to find the most important features while maximizing the model's effectiveness. Examples of regularizes include Lasso (L1), Ridge (L2), and Elastic Net (L3). These approaches penalize the values of feature coefficients during training, which promotes sparsity and the selection of relevant features. |
| Principal Component Analysis (PCA) | PCA is a dimensionality reduction method that converts the original features into a new set of uncorrelated components called principal components. The primary components are composed of the original traits and have the greatest impact on the data. By choosing a subset of the principal components, feature selection can be attained. |
| Feature Importance Methods | These methods determine the importance of features based on the impact that they have on the performance of a specific learning algorithm. Tree-based models, such as Random Forests, Extra Tree Classifiers, and Gradient Boosting Machines, can provide importance scores for features based on how frequently they are utilized for splitting and the degree to which they reduce the amount of impurity. These metrics can be employed to choose the most significant features. |
| Univariate Feature Selections | Univariate feature selection methods, such as Select-K-best, evaluate the association between each feature and the target variable separately. Statistical tests, such as t-tests or ANOVA, are employed to assess the degree to which each feature is significant about the target. Based on the p-values or other numerical measures, features are selected or prioritized. |
| Meta-Heuristic Methods | Meta-Heuristic Methods (e.g., Genetic Algorithms) are based on an evolutionary approach that seeks to find the optimal subset of features. They necessitate the creation of a population of feature subsets, evaluating their effectiveness using a fitness function (e.g., the accuracy of the model), and modifying the population over time via selection, crossovers, and mutations. Genetic Algorithms have a large search space and find a beneficial subset of features. Genetic Algorithms are particularly beneficial for problems that have a large, complex space of possible solutions, and multiple competing goals.  The key components of a Genetic Algorithm (GA) comprise:  Population (P): A collection of individuals, also known as chromosomes or solutions, each representing a potential solution to the problem at hand.  Individual (I): Each individual within the population represents a potential solution, typically encoded as a binary string where each bit corresponds to a gene or attribute of the solution.  Fitness Function (F): This function evaluates the quality or fitness of an individual by measuring its effectiveness in solving the problem. It maps each individual to a single numerical value representing its fitness.  Selection (S): The process of choosing individuals from the population to reproduce based on their fitness. Selection methods, such as roulette wheel selection, tournament selection, or rank-based selection, mimic natural selection by favouring individuals with higher fitness for the next generation.  Crossover (C): Involves combining genetic material from two parent individuals to create offspring, simulating genetic recombination in biological reproduction. This process typically involves selecting a crossover point and exchanging genetic material between parents to generate one or more offspring.  Mutation (M): Randomly altering the genetic composition of offspring to introduce diversity and explore different regions of the search space. Mutation prevents premature convergence by enhancing the algorithm's ability to explore. For instance, in binary encoding, mutation may involve flipping random bits within an individual's binary string.  Termination Criteria (TC): Determines when to halt the algorithm, often based on factors such as the number of generations, maximum evaluations per function, or a significant increase in fitness across iterations.  These components collectively drive the evolutionary process of Genetic Algorithms, facilitating the exploration and exploitation of the search space to discover optimal solutions. |
