# Supplementary material for: Stress management with HRV following AI, semantic ontology, genetic algorithm and tree explainer
Source: Sci Rep. 2025 Feb 17;15:5755. doi: 10.1038/s41598-025-87510-w (PMC11833117; doi:10.1038/s41598-025-87510-w)
Supplement: Supplementary file 3 — Supplementary Information 3. [file 41598_2025_87510_MOESM3_ESM.docx]

**Supplementary Table 3.** Different machine learning models in context.

| **Model Name** | **Description** |
| --- | --- |
| Logistic Regression (LR) | It is a statistical technique that models the probability of a dependent variable that is categorical or binary, based on one or more independent variables. It's effective for linearly separable data and is simple to comprehend, which makes it a popular choice for a variety of classification problems. |
| Linear Discriminant Analysis (LDA) | It is an unsupervised classification algorithm that attempts to find the linear combination of attributes that best separates the classes. Instead of modeling the probability of the dependent variable, it models the actual boundary between the classes directly. |
| K-Nearest Neighbors (KNN) | It is a non-parametric algorithm that predicts the class of new data based on the classes of its K-nearest neighbors in the training dataset. It's a simple and effective algorithm for small datasets; however, can be computationally intensive for large datasets. |
| Naïve Bayes (NB) | It is a probabilistic algorithm that assumes that the significance of a certain attribute in a class is independent of the presence of other attributes. It's straightforward, fast, and can work with high-dimensional data. |
| Decision Tree (DT) | It is a popular algorithm that creates a Decision Tree by recursively partitioning the data based on the most significant attribute. The Decision Tree can be employed to predict the class of a new data point by following the path from the root to the leaf node. Decision Trees are simple to comprehend and interpret, which makes them beneficial for a variety of classification issues. |
| Bagging Classifier | is a method of ensemble learning that involves multiple different models, typically trees of decision-making, being trained on different parts of the training data. Each model is dedicated to a random subset of the training data that is then replaced (bootstrap samples). The final estimate is derived by averaging the predictions of all individual models (for regression problems) or by majority rule (for classification problems). Bagging reduces variation and overfitting by including multiple models that are diverse. |
| Random Forest (RF) | It is a variation of bagging that employs decision trees as the primary models. Other than creating multiple trees of decision-making using bootstrap samples, Random Forest also incorporates a random selection of features at each split in the tree. This randomness helps to decouple the decision trees and promotes diversity in features, this leads to more accurate predictions. The final estimate in a Random Forest is derived through averaging or majority rule, like bagging. |
| Gradient Boosting (GB) | It is another ensemble method that combines multiple weak models (typically trees of decision-making) to create a powerful model. Unlike the bagging method, in which models are sequentially augmented with new features, boosting instead focuses on improving the quality of the previous model's features. Boosting increases the weight of samples that are misclassified and changes the model's parameters in subsequent steps to focus on them more. The final estimate is derived by combining the individual predictions that are weighted together. |
