# Supplementary material for: Stress management with HRV following AI, semantic ontology, genetic algorithm and tree explainer
Source: Sci Rep. 2025 Feb 17;15:5755. doi: 10.1038/s41598-025-87510-w (PMC11833117; doi:10.1038/s41598-025-87510-w)
Supplement: Supplementary file 4 — Supplementary Information 4. [file 41598_2025_87510_MOESM4_ESM.docx]

**Supplementary Table 4.** Different processes in machine learning pipeline design.

| **Process Name** | **Description** |
| --- | --- |
| Data Collection | We have collected publicly available SWELL-KS HRV datasets following the GDPR guidelines, and no identity has been disclosed. |
| Data Cleaning and Preprocessing | We have checked if there are any missing or NaN data values. We encoded categorical features. |
| Feature Engineering | We have used meta-heuristic genetic algorithm (GA) for feature optimization and to create an optimal feature set. |
| Data Splitting | We have used a ratio of 60:20:20 with shuffling, randomization, and stratification techniques to train, validate, and test ML models. |
| Model Selection | We have used traditional ML classifiers and selected the best classifier based on the metrics, such as accuracy, F1-score, precision, recall, MCC, and mean and standard deviation of the accuracies against cross-validation. |
| Model Training | We have used the Stratified K-Fold method and 5-fold cross-validation (Calibrated and Grid-Search Classifier CV) techniques. |
| Model Evaluation | We have compared and analyzed the individual ML model's validation curve, learning curve, and scalability. |
| Hyperparameter Tuning | We have used the traditional Grid-Search method for hyperparameter training. |
| Model Validation | We have used the 5-fold cross-validation (Calibrated and Grid-Search Classifier CV) techniques. |
| Model Deployment | We have prepared a pickle file to store the best-performing trained classifier models for the imbalanced and balanced datasets |
| Monitoring and Maintenance | We have executed all the above steps for both the imbalanced and balanced HRV datasets. |
