# Supplementary material for: Stress management with HRV following AI, semantic ontology, genetic algorithm and tree explainer
Source: Sci Rep. 2025 Feb 17;15:5755. doi: 10.1038/s41598-025-87510-w (PMC11833117; doi:10.1038/s41598-025-87510-w)
Supplement: Supplementary file 5 — Supplementary Information 5. [file 41598_2025_87510_MOESM5_ESM.docx]

**Supplementary Textbox 5.** High-level examples of the sample SPARQL queries for stress management based on HRV features.

| 1. SPARQL Query for Stress Management based on HRV Features.   PREFIX rdf: <http://www.w3.org/1999/02/22-rdf-syntax-ns#>  PREFIX rdfs: <http://www.w3.org/2000/01/rdf-schema#>  PREFIX owl: <http://www.w3.org/2002/07/owl#>  PREFIX hrv: <http://example.com/ontology/hrv#>  PREFIX stress: <http://example.com/ontology/stress#>  SELECT ?person ?stressLevel  WHERE {  ?person rdf:type stress:Person ;  stress:hasHRVMeasurement ?hrvMeasurement .  ?hrvMeasurement rdf:type hrv:HRVMeasurement ;  hrv:hasHRVFeature hrv:StressIndex ;  hrv:hasValue ?stressLevel .  FILTER (?stressLevel == 1) .  }   1. SPARQL Query for HRV Features.   PREFIX rdf: <http://www.w3.org/1999/02/22-rdf-syntax-ns#>  PREFIX rdfs: <http://www.w3.org/2000/01/rdf-schema#>  PREFIX owl: <http://www.w3.org/2002/07/owl#>  PREFIX hrv: <http://example.com/ontology/hrv#>  SELECT ?subject ?hrvFeature ?value  WHERE {  ?subject rdf:type hrv:HRVMeasurement ;  hrv:hasHRVFeature ?hrvFeature ;  hrv:hasValue ?value .  }   1. SPARQL Query to retrieve all HRV features.   PREFIX rdf: <http://www.w3.org/1999/02/22-rdf-syntax-ns#>  PREFIX rdfs: <http://www.w3.org/2000/01/rdf-schema#>  PREFIX owl: <http://www.w3.org/2002/07/owl#>  PREFIX hrv: <http://example.com/ontology/hrv#>  SELECT ?hrvFeature  WHERE {  ?hrvFeature rdf:type hrv:HRVFeature .  }   1. SPARQL Query to retrieve HRV features and their descriptions.   PREFIX rdf: <http://www.w3.org/1999/02/22-rdf-syntax-ns#>  PREFIX rdfs: <http://www.w3.org/2000/01/rdf-schema#>  PREFIX owl: <http://www.w3.org/2002/07/owl#>  PREFIX hrv: <http://example.com/ontology/hrv#>  SELECT ?hrvFeature ?description  WHERE {  ?hrvFeature rdf:type hrv:HRVFeature ;  rdfs:comment ?description .  }   1. SPARQL Query to retrieve HRV measurements with specific feature values.   PREFIX rdf: <http://www.w3.org/1999/02/22-rdf-syntax-ns#>  PREFIX rdfs: <http://www.w3.org/2000/01/rdf-schema#>  PREFIX owl: <http://www.w3.org/2002/07/owl#>  PREFIX hrv: <http://example.com/ontology/hrv#>  SELECT ?hrvMeasurement ?value  WHERE {  ?hrvMeasurement rdf:type hrv:HRVMeasurement ;  hrv:hasHRVFeature ?hrvFeature ;  hrv:hasValue ?value .  FILTER (?value > 0.6) .  } |
| --- |
