# Supplementary material for: Stress management with HRV following AI, semantic ontology, genetic algorithm and tree explainer
Source: Sci Rep. 2025 Feb 17;15:5755. doi: 10.1038/s41598-025-87510-w (PMC11833117; doi:10.1038/s41598-025-87510-w)
Supplement: Supplementary file 6 — Supplementary Information 6. [file 41598_2025_87510_MOESM6_ESM.docx]

**Supplementary Table 6.** A quantitative comparison table showcasing the novel contributions of our study compared to existing studies.

| **Study** | **Dataset** | **Features** | **Technique(s)** | **Metrics & score** | **Ethical AI** | **Semantics** | **X-AI** |
| --- | --- | --- | --- | --- | --- | --- | --- |
| Our study | SWELL-HRV | 10 | Genetic Algorithm + Random Forest (RF) + ADASYN | Accuracy = 99.8%, F1-score = 99.8%, Precision = 99.8%,  Recall = 99.8%, MCC = 99.1%, and standard deviation of accuracies = 0.0 | ✓ | ✓ | SHAP |
| Our study | SWELL-HRV | 8 | Genetic Algorithm + Random Forest (RF) + ADASYN | Accuracy = 99.8%, F1-score = 99.8%, Precision = 99.7%,  Recall = 99.8%, MCC = 99.1%, and standard deviation of accuracies = 0.0 | ✓ | ✓ | SHAP |
| Our study | SWELL-HRV | 10 | Genetic Algorithm + Random Forest (RF) + SMOTE | Accuracy = 99.8%, F1-score = 99.8%, Precision = 99.7%,  Recall = 99.9%, MCC = 99.1%, and standard deviation of accuracies = 0.0 | ✓ | ✓ | SHAP |
| Our study | SWELL-HRV | 8 | Genetic Algorithm + Random Forest (RF) + SMOTE | Accuracy = 99.8%, F1-score = 99.8%, Precision = 99.8%,  Recall = 99.6%, MCC = 99.1%, and standard deviation of accuracies = 0.0 | ✓ | ✓ | SHAP |
| Our study | SWELL-HRV | 10 | Genetic Algorithm + Random Forest (RF) | Accuracy = 99.4%, F1-score = 99.4%, Precision = 99.4%,  Recall = 99.4%, MCC = 98.8%, and standard deviation of accuracies = 0.0 | ✓ | ✓ | SHAP |
| Our study | SWELL-HRV | 8 | Genetic Algorithm + Random Forest (RF) | Accuracy = 99.4%, F1-score = 99.4%, Precision = 99.4%,  Recall = 99.4%, MCC = 98.8%, and standard deviation of accuracies = 0.0 | ✓ | ✓ | SHAP |
| Our study | SWELL-HRV | 34 | Random Forest (RF) | Accuracy = 99.9%, F1-score = 99.9%, Precision = 99.9%,  Recall = 99.9%, MCC = 98.4%, and standard deviation of accuracies = 0.0 | ✓ | ✓ | × |
| Muhajir et al. | HRV (not detailed) | 16 | ML | Accuracy = 70.0% | × | × | × |
| Giannakakis et al. | HRV (not detailed) | 16 | SVM | Accuracy = 84.4% | × | × | × |
| Liu et al. | HRV (not detailed) | Not specified | Spearman rank correlation, Bland–Altman plots | Accuracy = 85.3% | × | × | × |
| Can et al. | Self-reports, physiological measures | Not specified | SVM | Accuracy = 73.4% | × | × | × |
| Castaldo et al. | HRV (not detailed) | Not specified | ML | Accuracy = 80.0% | × | × | × |
| Hovsepian et al. | ECG, respiration | Not specified | SVM | Accuracy = 90.0% (lab), 72.0% (real-life) | × | × | × |
| Gjoreski et al. | Wearable device | Not specified | SVM | Accuracy = 92.0% | × | × | × |
| Schmidt et al. | HRV (not detailed) | Not specified | RF | Accuracy = 88.0% | × | × | × |
| Muaremi et al. | Biomarkers | Not specified | RF | Accuracy = 71.0% | × | × | × |
| Benchekroun et al. | MMSD, UWS | Not specified | Logistic Regression (LR), RF | F1-score; MMSD: 71.0%, UWS: 75.0% | × | × | × |
| Sriramprakash et al. | SWELL-KW | 17 | SVM | Accuracy = 92.7% | × | × | × |
| Sarkar et al. | SWELL-KW, AMIGOS | Not specified | Convolutional Neural Net (CNN) | Accuracy = 98.3% | × | × | × |
| Koldijk et al. | SWELL-KW | Not specified | SVM | Accuracy = 90.0% | × | × | × |
| Bobade et al. | WESAD | 7 | Artificial Neural Net (ANNs) | Accuracy = Binary: 84.32%, Multi-class: 95.21% | × | × | × |
| Arsalan et al. | WESAD | 7 | Multi-Layer Perceptron (MLPs) | Accuracy = Binary: 92.85%, Multi-class: 64.28% | × | × | × |
| Albaladejo et al. | SWELL-KW | 34 | MLPs | Accuracy = 88.64% | × | × | × |
| Mortensen et al. | SWELL-KW | 34 and 14 | 1D-CNN | Accuracy = All features: 99.99%, Optimized features: 96.5% | × | × | × |
| Ghose et al. | SWELL-KW | 3 | K-Nearest Neighbor (K-NN) | Accuracy = 99.3% | × | × | LIME |
